# Ethylene-mediated improvement in sucrose accumulation in ripening sugarcane involves increased sink strength

**DOI:** 10.1186/s12870-019-1882-z

**Published:** 2019-06-28

**Authors:** Zhongliang Chen, Cuixian Qin, Miao Wang, Fen Liao, Qing Liao, Xihui Liu, Yangrui Li, Prakash Lakshmanan, Minghua Long, Dongliang Huang

**Affiliations:** 1 College of Agriculture, Guangxi University, Nanning, 530004 China; 2 Key Laboratory of Sugarcane Biotechnology and Genetic Improvement (Guangxi), Ministry of Agriculture and Rural Affairs /Guangxi Key Laboratory of Sugarcane Genetic Improvement /Sugarcane Research Institute, Guangxi Academy of Agricultural Sciences, Nanning, 530007 China; 3 Queensland Alliance for Agriculture and Food Innovation, The University of Queensland, QLD, St Lucia, 4072 Australia

**Keywords:** Sugarcane, Ethylene, Transcriptomics, Sucrose accumulation

## Abstract

**Background:**

Sugarcane is a major crop producing about 80% of sugar globally. Increasing sugar content is a top priority for sugarcane breeding programs worldwide, however, the progress is extremely slow. Owing to its commercial significance, the physiology of sucrose accumulation has been studied extensively but it did not lead to any significant practical outcomes. Recent molecular studies are beginning to recognize genes and gene networks associated with this phenomenon. To further advance our molecular understanding of sucrose accumulation, we altered sucrose content of sugarcane genotypes with inherently large variation for sucrose accumulation using a sugarcane ripener, ethylene, and studied their transcriptomes to identify genes associated with the phenomenon.

**Results:**

Sucrose content variation in the experimental genotypes was substantial, with the top-performing clone producing almost 60% more sucrose than the poorest performer. Ethylene treatment increased stem sucrose content but that occurred only in low-sugar genotype. Transcriptomic analyses have identified about 160,000 unigenes of which 86,000 annotated genes were classified into functional groups associated with carbohydrate metabolism, signaling, localization, transport, hydrolysis, growth, catalytic activity, membrane and storage, suggesting the structural and functional specification, including sucrose accumulation, occurring in maturing internodes. About 25,000 genes were differentially expressed between all genotypes and treatments combined. Genotype had a dominant effect on differential gene expression than ethylene treatment. Sucrose and starch metabolism genes were more responsive to ethylene treatment in low-sugar genotype. Ethylene caused differential gene expression of many stress-related transcription factors, carbohydrate metabolism, hormone metabolism and epigenetic modification. Ethylene-induced expression of ethylene-responsive transcription factors, cytosolic acid- and cell wall-bound invertases, and ATPase was more pronounced in low- than in high-sugar genotype, suggesting an ethylene-stimulated sink activity and consequent increased sucrose accumulation in low-sugar genotype.

**Conclusion:**

Ethylene-induced sucrose accumulation is more pronounced in low-sugar sugarcane genotype, and this is possibly achieved by the preferential activation of genes such as invertases that increase sink strength in the stem. The relatively high enrichment of differentially expressed genes associated with hormone metabolism and signaling and stress suggests a strong hormonal regulation of source-sink activity, growth and sucrose accumulation in sugarcane.

**Electronic supplementary material:**

The online version of this article (10.1186/s12870-019-1882-z) contains supplementary material, which is available to authorized users.

## Background

Sugarcane (*Saccharum sp*. *L*.) is one of the most valuable sugar and bioenergy crops and is grown in at least 106 countries spread across tropics and sub-tropics [1]. Globally about 80% of sugar is produced from sugarcane. It has a remarkably high capacity for sucrose accumulation in the stem, reaching its level up to 0.7 M in mature internodes [2]. Sugarcane produces the greatest crop tonnage and is the second largest biofuel crop in the world [3, 4]. This large perennial C4 graminaceous plant is also one of the most genetically complex crops, making variety improvement through breeding slow and difficult.

Sucrose content is the most important commercial trait for sugarcane crop. From a commercial production perspective, the value of increasing sugar yield (t/ha) by improving sugar content in sugarcane stem is estimated to be ~ 1.8 times greater than an equivalent proportional improvement achieved through improved cane yield [5]. Because of the commercial significance, sugar content is a priority trait for all sugarcane variety improvement programs worldwide. However, despite extensive breeding effort, improvement in sucrose content remains very slow globally, and in some countries, it has been plateaued for a long time [1]. Recognising the difficulty of sucrose content improvement through conventional means, substantial resources have been directed to understand the physiological, cellular, biochemical and molecular bases of sugar production and accumulation in sugarcane with the ultimate objective of improving sugar content by molecular or a combined molecular and conventional strategies [6–11]. These studies, though mostly conducted with a single sugarcane genotype, isolated tissues, cell cultures and protoplasts, greatly expanded our understanding of sucrose metabolism pathways and enzymes involved, sucrose transporters and some of the physiological factors influencing sucrose accumulation. They also helped develop a few simple but logical models of sucrose accumulation in sugarcane [2, 7, 12]. However, experimental validation of these models to identify and potentially remove the key bottlenecks of sucrose accumulation, including using reverse genetics strategies, fell short of expectation.

The consequent realization that sucrose synthesis and accumulation in sugarcane is highly complex and that it may involve a large network of interactions operating at different levels of organization led to studies aimed at understanding the dynamics of gene and metabolic activities associated with sucrose accumulation at a more global scale [8, 13, 14]. The strong evidence of sucrose as a signaling molecule modulating a myriad of growth and developmental processes including cell division, cell differentiation and accumulation of storage products further strengthened this strategy [15, 16]. Microarray, transcriptomics and metabolomics studies on sucrose synthesis and accumulation in sugarcane internodes, though very limited in numbers, reported an association of sucrose level and the activity of a network of genes related to cell wall synthesis, stress responses, flowering, carbohydrate metabolism, lignin synthesis and sugar transport [8, 13, 14, 17, 18]. Most of these investigations compared low and high sugar varieties and generated considerable data and useful hypotheses for future investigation. For instance, Casu et al. (2005) [13] after extensive EST analyses concluded that sucrose accumulation in sugarcane is regulated by a network of growth and developmental processes associated with stem maturation, which include fibre production, vacuole formation, transport processes and osmotic changes. In another study, Iskandar et al. (2011) [19] altered sugar production in sugarcane stem by imposing water stress and observed no correlation between water stress-induced gene expression and sucrose accumulation. A more recent research on transcriptional basis of sucrose accumulation found a large set of genes differentially expressed between high and low sugar genotypes and proposed more studies to discern whether the sucrose level regulates the observed gene expression or vice versa [8]. Clearly, the molecular regulation of this unusual phenomenon of sucrose accumulation to extremely high levels of sucrose in sugarcane stem is far from understood.

Use of an appropriate experimental model greatly facilitates mechanistic studies of physiological processes such as sucrose accumulation in sugarcane stem. Crop production strategies aimed at improving stem sugar content by reducing vegetative growth and thereby channeling photosynthetically fixed carbon for sucrose accumulation in sugarcane have been in practice for a long time in many countries. This include withdrawing irrigation several weeks prior to harvest and the use of chemicals, primarily plant growth regulators, as ripening agents [20–23]. Ethylene-releasing compound ethephon was the first chemical ripener commercially used in sugarcane crops, and, as with most ripeners, the response to ethephon is genotype-dependent [22, 24]. The retention of sucrose accumulated in response to ripener application in sugarcane depends on the genotype and the growing condition of crop following ripener treatment [25]. The biochemical or molecular mechanism(s) underpinning this genotype-specificity and how ethylene enhances sucrose accumulation in sugarcane remain unclear. A previous study on ethylene-mediated sucrose accumulation using young sugarcane plants grown in a glasshouse has identified a reduction in leaf size and an increase in specific leaf area resulting in greater diversion of photosynthate to sink tissue (stem), accelerated internode formation and increased number of internodes as the contributing factors for increased sucrose production in ethylene-treated plants [18]. The only published research on combined transcriptional and hormonal changes in ethylene-treated mature field-grown sugarcane plants of a single commercial variety highlights an association between ethylene-induced sucrose accumulation, increased defense response and inactivation of auxins and gibberellins during ripening [26]. In this study reported here, we used ethylene-induced sucrose accumulation in the field as an experimental system to expand our molecular understanding of sucrose accumulation in sugarcane occurring naturally (natural ripening) and in response to ethylene application (accelerated ripening) in genotypes with large variation for sugar content. In responsive genotypes, ethylene boosts sucrose accumulation considerably and consistently compared to other ripeners, making the experimental system a robust tool for studying sucrose accumulation in sugarcane. Here, we conducted detailed transcriptome and sucrose analyses and identified patterns of gene expression and genes associated with sucrose accumulation, providing an explanation for differential genotypic response of ethylene-induced sucrose accumulation during accelerated ripening.

## Results

### Ethephon-induced sucrose production occurred only in the low-sugar clone

**Fig. 1:**
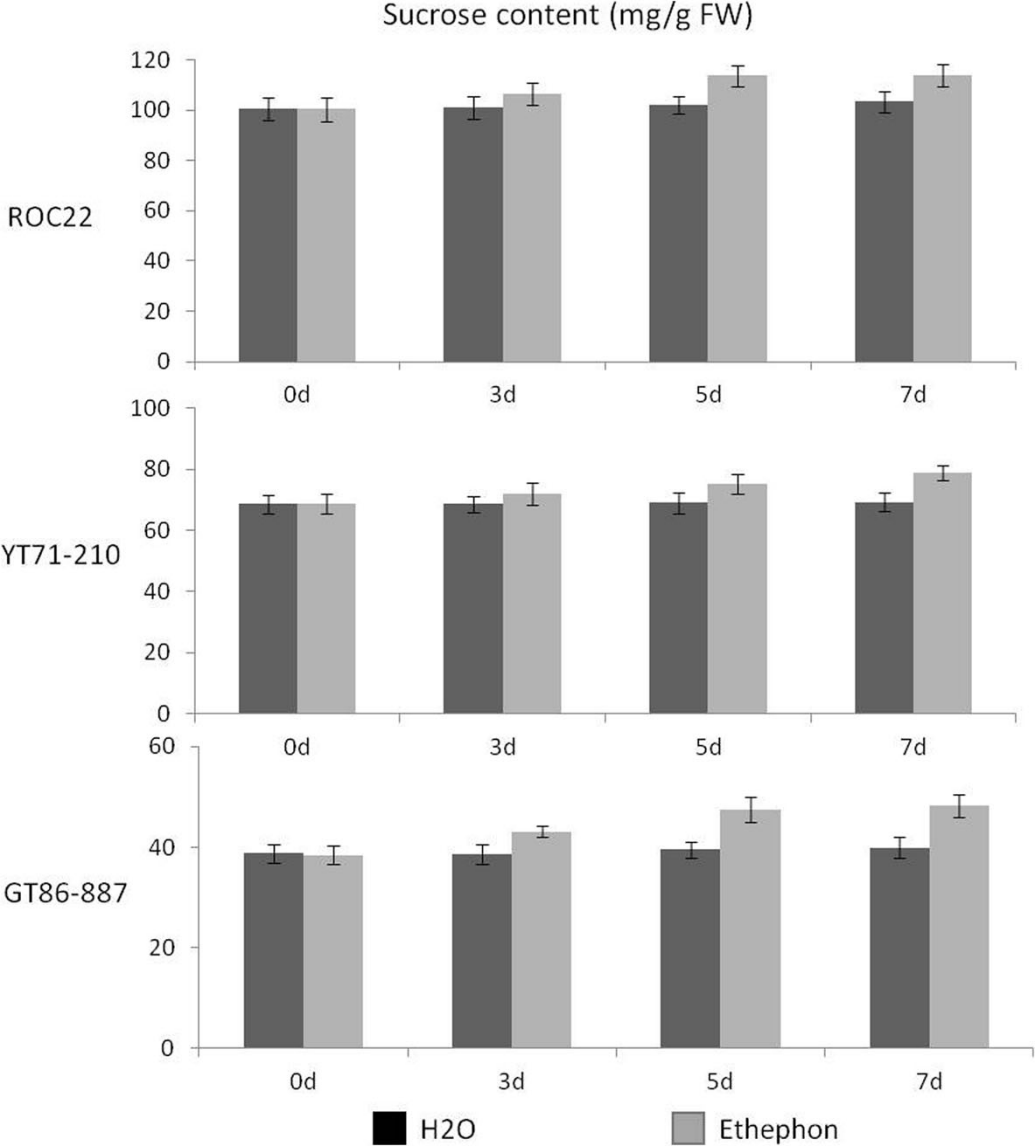
Effect of ethylene on sucrose content in mature stems of three sugarcane genotypes, ROC22, YT71–210 and GT86–887. Stem sugar content was quantified before (0 d) and 3, 5 and 7 days after ethylene treatment. The control treatment received water. The ordinate headline shows the ratio of sugar content to fresh weight (mg/g) ± SD. Data were analysed by ANOVA following a repeated measurements model using Genstat (VSNC, UK). Genotype (LSD 3.4) and treatment (LSD 2.8) effects are highly significant (*p* < 0.001) for day 5 and day 7

The analysis of sucrose content data showed highly significant (*P* < 0.001) genotype and treatment effects but there was no genotype by treatment interaction (Fig. 1). The top-performing high-sugar clone (HS) ROC22 produced almost 60% more sucrose (103 mg/g FW) than the low-sugar clone (LS) GT86–887 (40 mg/g FW) in the control treatment (Fig. 1). External application of ethephon increased sugar content by about 20% in GT86–887 (P < 0.001) as compared to control on day five after treatment, but it failed to elicit any difference in sucrose content in ROC22 and medium-sugar clone (MS) YT71–210 during that period (Fig. 1). The variation for sugar content between treatments did not change on day seven, the last point of measurements, in all the genotypes.

### Transcriptome analysis and identification of unigenes

To better understand the molecular changes associated with the natural variation for sucrose accumulation occurring in sugarcane, and how that process is affected by the externally supplied ethephon, we analysed transcriptome of young developing parts of sugarcane stem (developing/maturing phytomers) from ethephon-treated and control plants from all three genotypes using Illumina paired-end HiSeq platform. Samples from two biological repeats for each treatment from each genotype were prepared and sequenced (Additional file 11: Table S1).

The reproducibility of the sequencing data was evaluated by Pearson’s correlation coefficients and it was calculated by log_10_ (RPKM+ 1). All biological replicates were strongly correlated (R^2^ ≥ 0.90) with each other (Additional file 1: Figure S1). After quality assessment and data clearance, a total of more than 3 billion (G) reads combined from all samples were retained as high-quality reads and used in subsequent analyses (Additional file 11: Table S1).

**Fig. 2:**
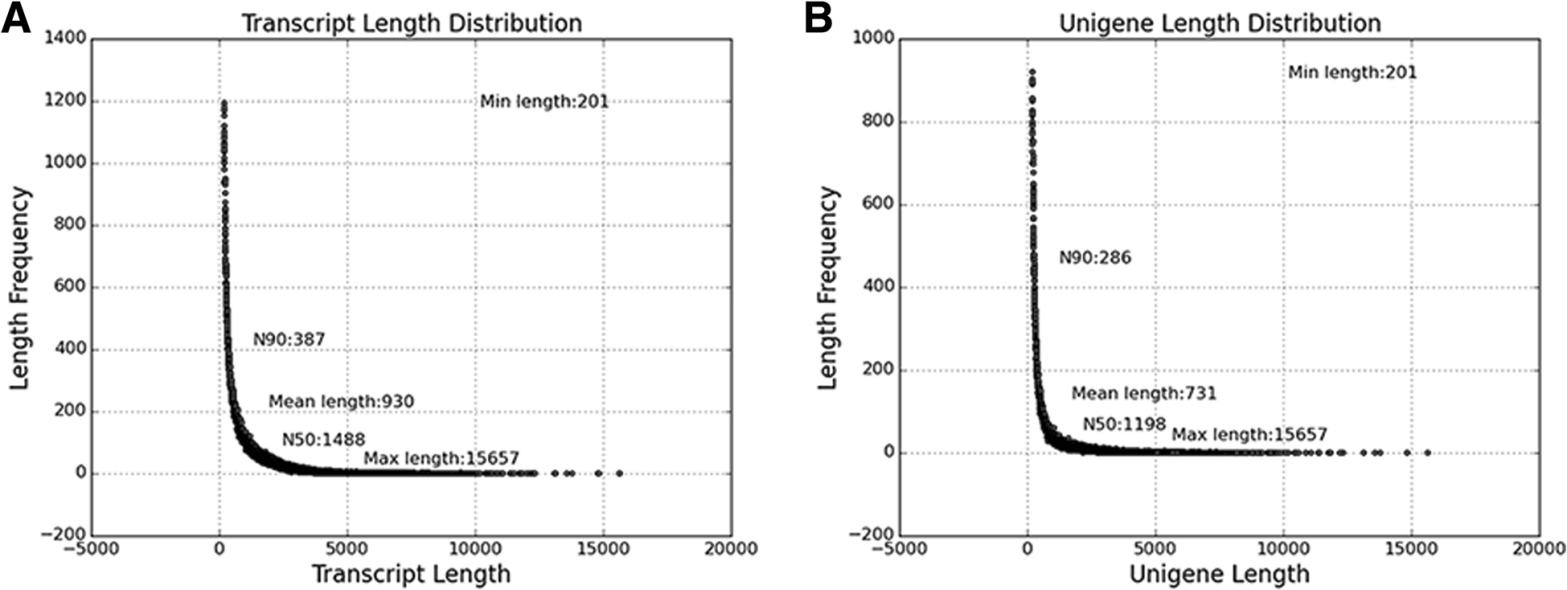
Statistics of transcripts and unigenes obtained from RNA-seq data. **a** Transcripts assembled from the RNA-seq data. **b** Unigenes obtained from the transcripts assembled

The transcriptome assembled from the RNA-seq data was used as reference. The assembly of the clean reads was carried out using Trinity [27] and they were analyzed (Additional file 12: Table S2 and Additional file 13: Tables S3). A total of 337,456 transcripts with an average length of 930 bp and N50 of 1488 were assembled by de novo assembly. From this assembly 163,054 UniGenes with an average length of 731 bp and N50 of 1198 bp were identified (Fig. 2 and Additional file 2: Figure S2).

The unigenes were annotated in databases including NR, NT, KO, SwissProt, PFAM, GO, and KOG. Among them, 86,944 unigenes could be annotated through at least one database used, whereas 8385 of them were annotated in all databases (Additional file 14: Table S4). About 32% of the unigenes were annotated by Gene Ontology (GO) analysis, and were divided into three functional categories: those associated with biological processes, cellular components and molecular functions (Additional file 3: Figure S3A). For the “biological process” group, the most frequently associated terms were growth, development, metabolism, regulation, signailing, localisation and tissue organisation processes. For the “cellular component” group, the dominant classes were related to cell compartments, macromolecules, membranes, organelles and cellular structural parts. Catalitycal activity, binding, transport, macromolecular processes, molecular transducers and transcription factor were the major classes in the “molecular function” grouping. About 10% of the unigenes were annotated by KAAS to obtain the corresponding KO numbers, which were used to annotate them in the KEGG pathway (Additional file 3: Figure S3B). Based on the KO results, the genes were separated into 5 clusters in which the first three bins were involved in signal transduction, translation and carbohydrate metabolism, with 1782, 1886 and 1774 genes, respectively. Using KOG, 12% of the identified unigenes were annotated (Additional file 3: Figure S3C), and they were divided into 26 groups, with general function prediction only, translation and ribosomal biogenesis, post-translational modifications and protein turnover, signal transduction and energy production and conversion being the dominant ones.

We further analyzed the specific distribution of unigenes (67147) annotated with NR database based on BLASTx, which revealed that the transcripts from sugarcane share high sequence similarity with that of *Sorghum bicolor* (41.5%), *Zea mays* (20.1%), *Setaria italica* (8.8%), *Oryza sativa* (5.3%), and *Oncorhynchus mykiss* (5.3%) (Additional file 3: Figure S3D).

### Genotype had a dominant effect on differential gene expression than ethylene treatment

Among the annotated genes, approximately 70,000 genes were expressed in each of the samples, and 38,692 genes were co-expressed in all of the samples (Additional file 1: Figure S1A). To gain more insights into the molecular changes associated with the intrinsic genotypic and ethylene-induced variation for sucrose accumulation, transcriptomes of three genotypes with and without ethephon treatment were screened for differentially expressed genes (DEGs) by pairwise comparisons.

There were 24,938 genes differentially expressed in all of the samples combined. We performed hierarchical clustering of these DEGs using the Euclidean distance method associated with complete-linkage. The expression patterns of six clusters, subcluster1–subcluster6, were plotted (Additional file 4: Figure S4). In LS clone, 4889 genes were down-regulated and 11,806 genes were up-regulated irrespective of the treatment (Additional file 4: Figure S4 subcluster1 and 2), among which 1154 genes were up-regulated more than five-fold (Additional file 4: Figure S4 subcluster5). In both LS and HS genotypes, 2182 genes were down-regulated and 5177 genes were up-regulated (Additional file 4: Figure S4. Subcluster 3 and 4). One cluster involving 450 genes were up-regulated in all the samples except the MS genotypes under ethylene treatment (Additional file 4: Figure S4 subcluster 6).

**Fig. 3:**
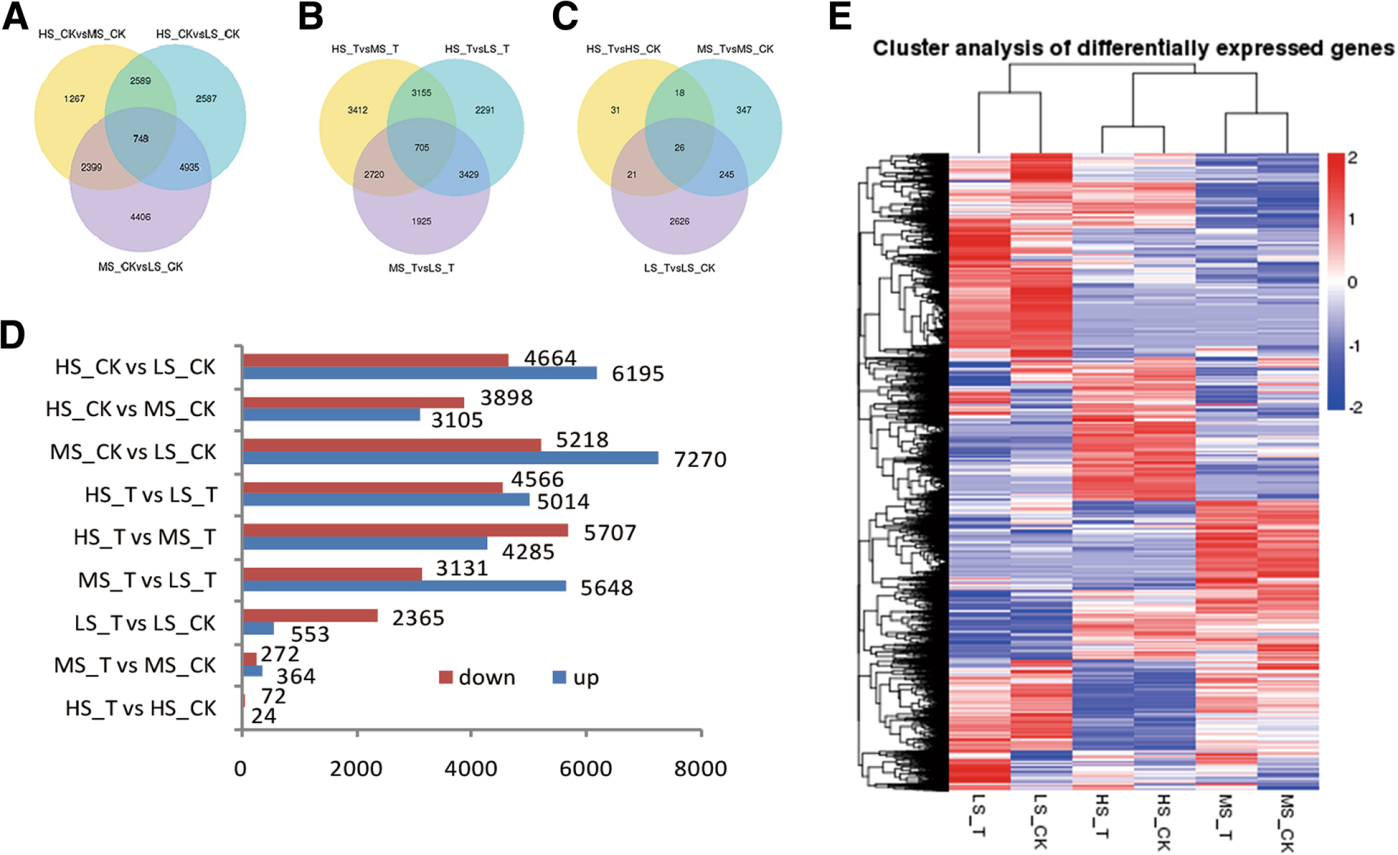
Statistics of DEGs identified in maturing stem tissue in different sugarcane genotypes treated with ethephon. Venn diagram of pairwise comparisons DEGs in three genotypes (**a**) before ethephon treatment (**b**) after ethephon treatment and (**c**) before and after ethephon treatment. (**d**) Number of up-regulated and down-regulated DEGs before and after ethephon treatment in 3 genotypes. (**e**) Heatmap of DEGs in 3 genotypes before and after ethephon treatment. Expression values are presented as RPKM normalized log_2_ transformed counts. Red and blue colors indicate up- and down- regulated transcripts, respectively. CK- check (water control), T- ethephon treatment, HS: high-sugar variety, MS: medium-sugar variety, LS: low-sugar variety

Pair-wise comparisons of the three genotypes without ethylene treatment, MS_CK vs. LS_CK, HS_CK vs. MS_CK and HS_CK vs. LS_CK, revealed 12,488, 7003 and 10,859 DEGs, respectively, with 748 DEGs being common to all (Fig. 3a). In the same three genotypes following ethylene treatment, the comparison of transcriptomes of MS_T vs LS_T, HS_T vs MS_T and HS_T vs LS_T revealed as many as 8779, 9992 and 9580 DEGs, respectively (Fig. 3b). Transcriptome comparison of the same genotype with and without ethylene treatment, MS_T vs. MS_CK, LS_T vs. LS_CK and HS_T vs. HS_CK, identified 636, 2918 and 96 DEGs, respectively (Fig. 3c). The number of up-regulated DEGs was larger than that of down-regulated ones in LS_T vs. LS_CK, HS_T vs. MS_T, HS_CK vs. MS_CK and HS_T vs. HS_CK comparisons, but the opposite was true for MS_T vs. MS_CK, MS_T vs. LS_T, MS_CK vs. LS_CK, HS_T vs. LS_T and HS_CK vs. LS_CK (Fig. 3d). Overall the data on gene expressions shows genotype-dependent ethylene response, similar to sugar production. The most dramatic expression changes were found in the comparison between HS_CK and LS_CK, which indicate the occurrence of significant gene level variation for sugar production among different sugarcane genotypes. Further, the heatmap on gene expression profiles (Fig. 3e), revealed that more significant gene expression differences exist between genotypes than between treatments.

### Sucrose and starch metabolism genes were expressed more abundantly in high-sugar than in low-sugar genotype, but they were more responsive to ethylene treatment in low-sugar genotype

Classification of DEGs based on GO annotations helped to group them into different functional categories. This was followed by pairwise comparisons of samples between genotypes grown under the same condition, and between samples from the same genotype grown under different conditions. The over-represented GO terms of DEGs were in three GO categories (cellular component, molecular function, and biological processes) (Additional file 5: Figure S5). In the molecular function category, most GO terms such as catalytic activity, transcription factors, transport, binding, etc., were enriched significantly in most pairwise comparisons except for HS_T vs. HS_CK and MS_T vs. MS_CK. Also, the terms of “Kinase activity”, “Phosphotransferase activity” and “Energy metabolism” were enriched significantly in this group (Additional file 5: Figure S5A). In the biological process category, GO terms of” Phosphorylation”, “Protein modification” and “Stress responses “were enriched significantly in most comparisons (Additional file 5: Figure S5B). In the cellular component category, no GO term was enriched significantly in comparisons between different genotypes. However, some terms such as apoplast, cell periphery and nucleoplasm were enriched in samples from ethylene treatment which suggested the effect of ethylene on the cellular components and activities in apoplasm (Additional file 5: Figure S5C).

KEGG analysis was also performed to explore the pathways in which DEGs are involved (Additional file 6: Figure S6). The DEGs were assigned to photosynthesis, plant hormone signal transduction, plant-pathogen interaction, starch and sugar metabolism, stress responses, lipid metabolism, apoptosis and amino-sugar and nucleotide-sugar metabolism. The most co-expression genes found among the different pairwise comparisons were involved in plant hormone signal transduction and plant-pathogen interaction. According to the GO and KEGG analysis, DEGs were further clustered between different genotypes irrespective of the treatment condition.

**Fig. 4:**
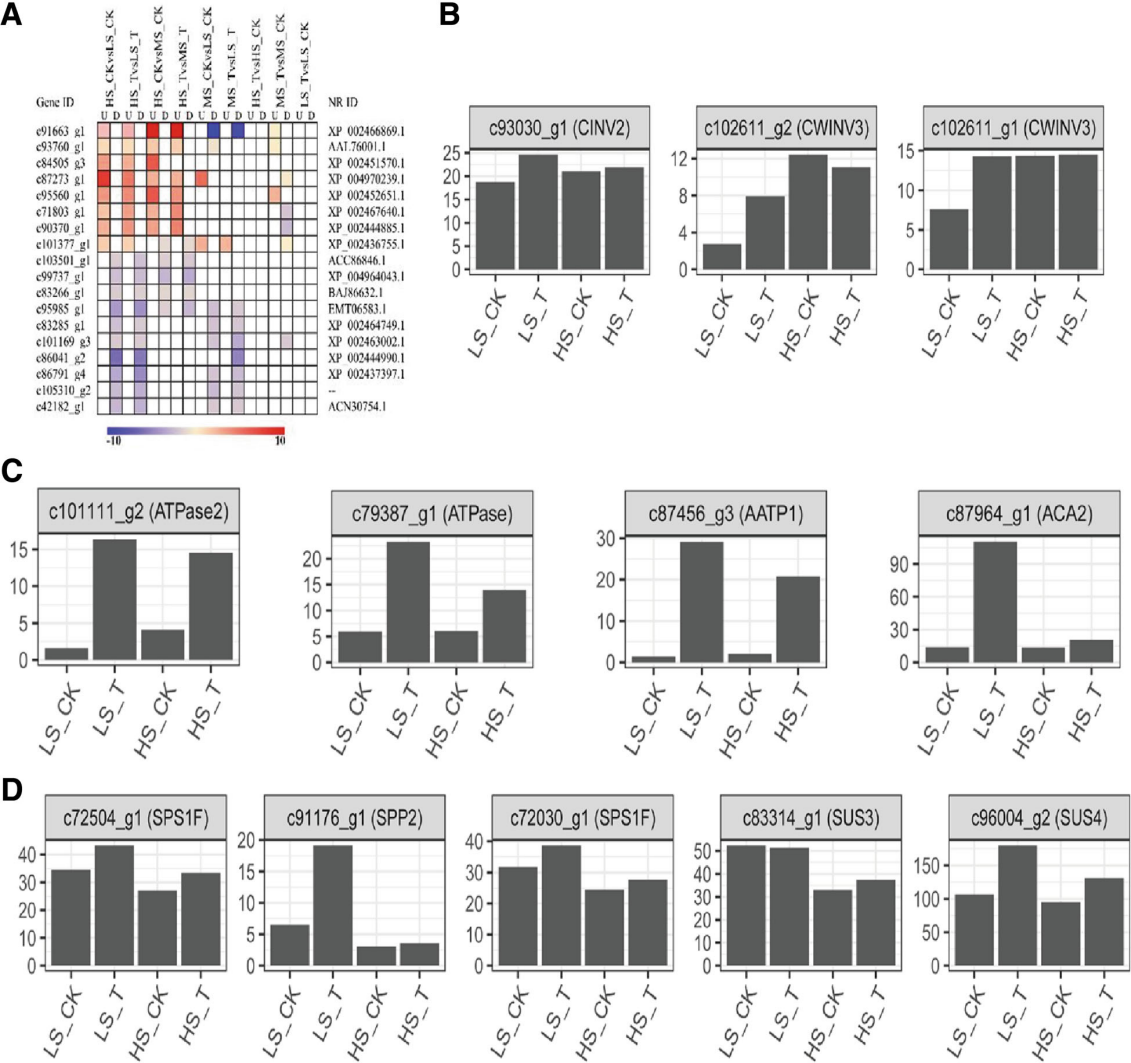
Analysis of differential expression of unigenes associated with carbohydrate metabolism. Pairwise comparison of differentially expressed unigenes involved in starch and sucrose metabolism (**a**). The significance in each comparison pair is indicated using log-transformed *P*-value (red); the dark gray areas represent missing values. Expression of invertases (**b**), ATPase (**c**) and SPS/SUS in HS and LS (**d**) as affected by ethylene treatment. CK- check (water control), T- ethephon treatment, HS: high-sugar variety, MS: medium-sugar variety, LS: low-sugar variety

Evidence published so far indicate the existence of a complex mechanism to control sugar metabolism and accumulation in sugarcane. To further understand the gene regulatory dynamics of sucrose production in sugarcane, DEGs involved in *carbohydrate* metabolism were selected for pairwise comparisons (Fig. 4a). The results showed that under both water and ethylene treatments, the expression of unigenes involved in starch/sucrose metabolic pathway, such as *c91663_g1* (sucrose α-glucosidase, INV), *c93760_g1* (sucrose α-glucosidase, INVs), *c84505_g3* (sucrose α-glucosidase, INV), *c87273_g1* (polygalacturonase) and *c95560_g1* (6-phosphofructokinase), and cell wall modifying genes *c71803_g1* (pectinesterase) and *c90370_g1* (pectinesterase) was higher in HS than that in MS and LS. The transcript of *c101377_g1* (starch synthase) in HS and MS was higher than that of LS. Some other unigenes including *c83285_g1*, *c101169_g3* (cellulose synthase), *c86041_g2* (1,3-beta-D-glucan synthase), *c86791_g4* (phloem sucrose loading), *c105310_g2* (1,3-beta-D-glucan synthase), *c42182_g1* (1,4-alpha-glucan branching enzyme) were highly expressed in HS and MS. These highly expressed starch/sucrose metabolism related genes suggest the occurrence of high starch/sucrose metabolic strength in HS compared to MS and LS. Also, there were other unigenes highly expressed in MS and LS, which include *c103501_g1* (starch synthase), *c99737_g1* (sucrose alpha-glucosidase), *c83266_g1* (pectinesterase), and *c95985_g1* (phosphotransferase) with function mostly in starch and sucrose metabolism and cell wall modification.

We compared the differentially expressed starch and sucrose metabolic pathway (KEGG) in HS and LS. Many genes involved in the starch and sucrose metabolism were changed. The *amylase* (EC3.2.1.1 and EC3.2.1.2), *starch synthase* (EC2.4.1.31) and *invertase* (EC3.2.1.26) were more intensely expressed in HS while the expression of *hexokinase* (EC2.7.1.1), *glucan-branching enzyme* (EC2.4.1.18) and *glycogen-branching enzyme* (TreXYZ) was lower (Additional file 7: Figure S7). Also, the genes involved in Photosystem I, Photosystem II and related protein complexes were differentially expressed in HS and LS (Additional file 8: Figure S8).

Sugar transport is an essential process for sugar accumulation in sugarcane [28–30]. We analyzed the expression of sugar transporter genes. Cytosolic and cell wall invertases are supposed to function in sugar transport through symplast and apoplast [31–34]. From the transcriptome data, the cytosolic acid invertase *c93030_g1* (annotated as CINV2) and cell wall invertases *c102611_g1* (CWINV3) and *c102611_g2* (CWINV3) were all expressed in both HS and LS, and the expression of *CWINV3* was significantly high in HS and enhanced by ethylene treatment in LS (Fig. 4b). Meanwhile, the cellular pH and activity of ATPases affect sugar transport through the membrane. Expression of *c101111_g2* (ATPase2), *c79387_g1* (ATPase), *c87456_g3* (AATP1) and *c87964_g1* (ACA2) were significantly induced by ethylene treatment (Fig. 4c), which may be part of the mechanism by which ethylene increases sucrose accumulation in sugarcane.

The sucrose biosynthesis in the source organ (leaf) also affects its accumulation in sugarcane stem. Starch phosphorylase (SPase) and sucrose phosphate synthase (SPS) are the two key enzymes in sucrose synthesis. SPS catalyzes the binding of UDPG to F6P to form sucrose phosphate and ethylene strongly stimulated SPS transcript accumulation in banana [35]. The expressions of *c72504_g1* (SPS1F), *c91176_g1* (SPP2), *C72030_g1* (SPS1F) displayed no significant difference in HS and LS, while they were all influenced by ethylene treatment in LS (Fig. 4d, Additional file 7: Figure S7). SuSy catalyses sucrose biosynthesis or degradation depended on the availability of UDP-Glucose [36, 37]. Relatively higher expression of *c83314_g1* (SUS3) and *c96004_g2* (SUS4) were recorded in LS than in HS and their expression was induced by ethylene treatment (Fig. 4d). We also analyzed the impact of ethylene treatment on expression of photosynthetic genes and found that only two proteins in Photosystem II were affected by ethylene (Additional file 8: Figure S8). Activity of these genes, however, did not display obvious correlation with the sugar content in the test plants.

### Ethylene caused differential expression of many transcription factors related to abiotic stress, carbohydrate metabolism, hormone metabolism and signaling, and epigenetic modifiers

**Fig. 5:**
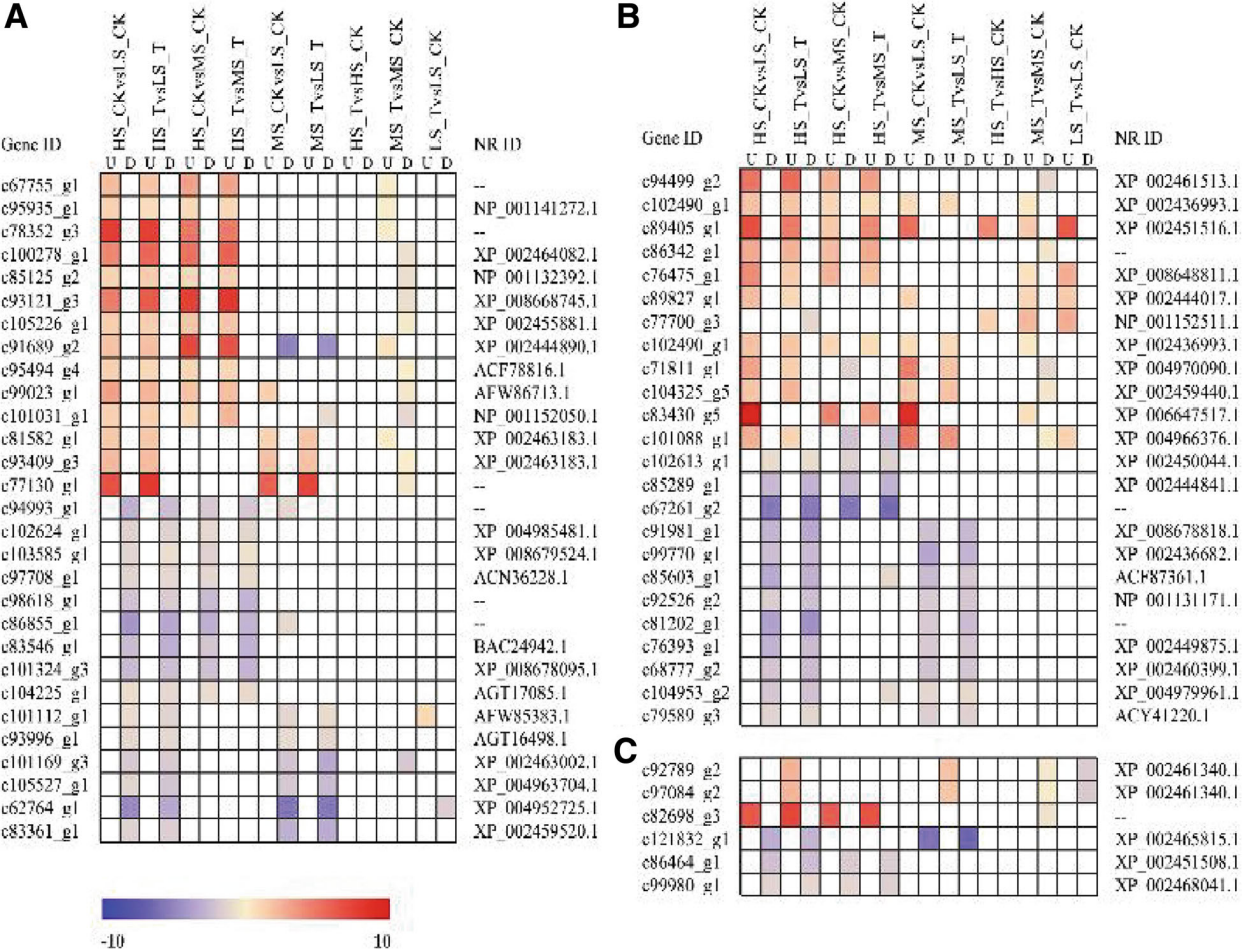
Analysis of differential expression of unigenes associated with transcription. Pairwise comparison of differentially expressed unigenes involved in transcriptional regulation (**a**), phosphorylation (**b**) and DNA methylation (**c**). The significance in each comparison pair is indicated using log-transformed *P*-value (red); the dark gray areas represent missing values. CK- check (water control), T- ethephon treatment, HS: high-sugar variety, MS: medium-sugar variety, LS: low-sugar variety

To further understand the regulatory network of carbohydrate metabolism as affected by ethylene treatment, expression patterns of transcription factors, regulatory proteins as well as the epigenetic factors were analyzed. Genes with the similar expression pattern in at least two pairwise comparisons were selected for further studies. Expression profiles of the genes annotated as transcription factor are shown in Fig. 5a. The expression of transcription factors (TFs) such as *c67755_g1*, *c95935_g1*, and *c78352_g3* (sequence-specific DNA binding TFs, sugarcane symporter TFs, Zinc ion binding, etc.) was higher in HS than in MS and LS irrespective of the treatment, and they were only slightly up-regulated by ethylene treatment in MS. Similarly, expression of transcription factors *c100278_g*1, *c85125_g2*, *c93121_g3*, *c105226_g1*, *c95494_g4*, *c99023_g1* and *c101031_g1* was higher in HS than in MS and LS, but they were down-regulated following ethylene treatment in MS. Transcription factors *c81582_g1*, *c93409_g3* and *c77130-g1* also showed higher expression in HS and MS than LS whereas an opposite trend was observed for transcription factors *c94993_g1*, *c102624_g1*, *c103585_g1*, *c97708_g1*, *c98618_g1*, *c86855_g1*, *c83546_g1*, *c101324_g3*, *c104225_g1* (TFs associated with protein catabolism, sequence-specific DNA binding, WRKY DNA binding domain, etc.) irrespective of the growing condition. Transcription factors *c101112_g1, c93996_g1, c101169_g3, c105527_g1, c62764_g1* and *c83361_g1* (TFs associated with ATP binding, protein phosphorylation, cell cycle transcriptional regulation, ERFs, etc.) expressed low in HS and MS than LS, and they were down-regulated by ethylene.

Furthermore, the genes involved in histone modification, protein phosphorylation and DNA methylation modification with similar expression profiles were shown in Fig. 5b and c. Most of the genes annotated were shown to be involved in histone methylation or RNA-directed DNA methylation pathways. Several methyltransferases (e.g. *c94499_g2, c86342_g1, c102490_g1* and *c83430_g5*) were expressed highly in HS or HS and MS than that in LS. The expression of some of them (e.g. *c89405_g1*) was further induced by ethylene treatment. In contract some methyltransferases such as *c102613_g1, c85289_g1* and c*67261_g2* had lower expression in HS than in MS and LS, while *c91981_g1, c99770_g1*, *c85603_g1, c92526_g2, c81202_g1, c76393_g1, c68777_g2, c104953_g2* and *c79589_g3* showed higher expression in LS than in HS and MS. Similarly, as shown in Fig. 5c, another DNA modification enzymes N-acetyltransferase and histone acetyltransferases (e.g. *c82698_g3, c121832_g1, c86464_g1* and *c99980_g1* showed differential expression in all three test clones and in response to ethylene treatment with no clear trend in relation to sucrose accumulation.

**Fig. 6:**
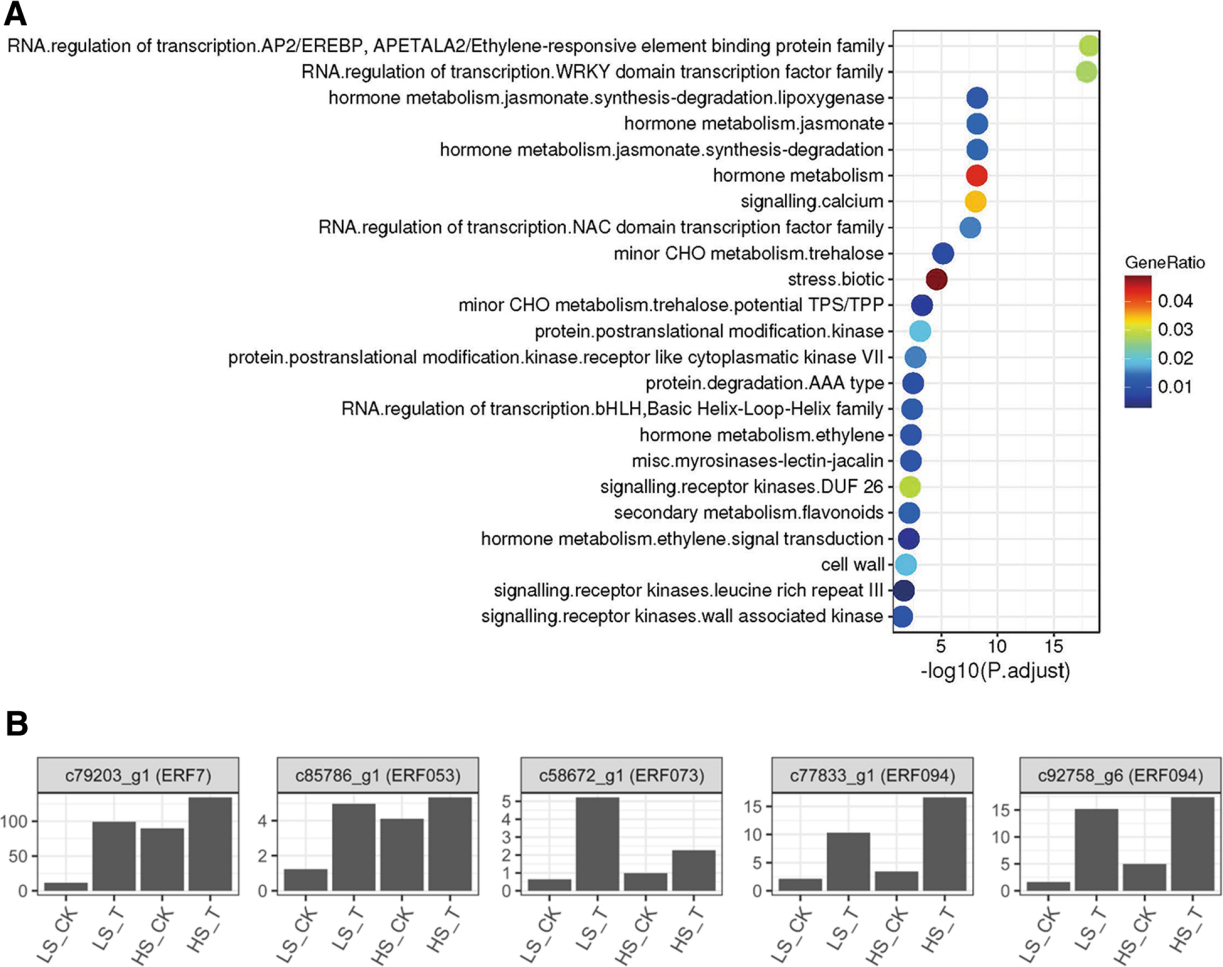
Functional enrichment analysis of differentially expressed genes in low-sugar (LS) sugarcane before and after ethylene treatment. **a** The up-regulated functions are displayed with respect to their significance (*p* < 0.01). Different colors correspond to the ratio of genes in each category. It’s noteworthy that the most enriched function is RNA-related transcription (corresponding to ethylene response factor; ERFs) and hormone-related metabolism and signaling (ethylene-related). These enriched modules will guid the search for the functional networks underpinning ethylene-regulated sugar production/translocation in sugarcane. **b** the expression of ERFs in HS and LS following ethylene treatment. CK- check (water control), T- ethephon treatment, HS: high-sugar variety, MS: medium-sugar variety, LS: low-sugar variety

The DEGs were further analyzed with MapMan, which classified the DEGs into 23 categories. Among them, the most enriched groups were those associated with RNA, regulation of transcription, ERFs/WRKYs, NAC domain transcription factors and hormone metabolism and signaling (Fig. 6a). Among the differentially expressed transcription factors, unigenes *c101031_g1, c97708_g1* and *c81582_g1* were annotated (NR database) as heat shock factor (HSF), WRKY and ethylene responsive factor (ERF), respectively. By comparing the expression of ERFs in the HS and LS varieties before or after the ethylene treatment, we found that most ERFs were expressed more abundantly in HS than in LS under normal condition, but the relative increase in their activity in response to ethylene treatment was much pronounced in LS varieties than in HS clone (Fig. 6b).

### Validation of candidate gene expression and identification of genetic elements associated with sucrose accumulation

According to the results of gene annotation and the gene expression, the genes involved in starch metabolism and sugar transport (*c91663_g1, c95560_g1, c71803_g1, c83266_g1, c93760_g1, c42181_g1*), transcription regulation factors (*c81582_g1, c93121_g3, c77130_g1, c101169_g1 and c83361_g1*), and the epigenetic modification (*c99770_g1*) were further analyzed by qRT-PCR (Additional file 9: Figure S9). The expression patterns of these genes in different genotypes were similar to the transcriptome results. Transcription regulation factors *c81582_g1, c93121_g3,* and *c77130_g1* showed relatively higher level of activity in HS clone ROC22 compared with that in YT71–210 (MS) and GT86–887 (LS). Only the transcript of ERF protein, *c81582_g1* increased in response to ethylene treatment in all the three genotypes suggesting its likely involvement in ethylene-mediated sugar accumulation or related growth processes. Starch metabolism and sugar transport-related genes *c91663_g1, c95560_g1, c71803_g1* were expressed remarkably high in HS clones and their activity was greatly inhibited by applied ethylene without any impact on sugar content.

The expression levels of *c83266_g1*, a gene involved in glucose metabolism, a transcription factor *c101169_gl* and an epigenetic modifier *c99770_g1* with protein-glutamate methylesterase were decreased with an increase in sugar content in 3 genotypes in the control. The transcript level of those genes, however, decreased remarkably in all three genotypes following ethylene treatment except for *c101169_gl* in the medium sugar clone YT71–210. These genes may be acting as negative regulators of sugar accumulation. The high proportion of *c91663_g1, c95560_g1* and *c71803_g1* transcripts, annotated to be involved in starch metabolism and sugar transport, in ROC22 than the other two genotypes in the control may also account for the observed genotypic difference in sugar content.

## Discussion

Sucrose accumulation in sugarcane is a complex process [2, 7, 9, 12, 38–40] and studying this phenomenon at molecular level is further complicated by the genetic complexity of the crop and the fact that genes associated with sucrose metabolism were indeed not abundantly expressed in stem tissues [13, 14]. As Watt et al. (2005) [14] noted, the premise that identifying genes expressed in stem tissues at different developmental stages would lead to the identification of genes regulating sucrose accumulation did not achieve the desired outcome. This is largely a corollary of formulating genomics strategies in isolation from the extensive physiological, biochemical and modelling data available on sucrose accumulation in sugarcane and other plants. Hence, exploiting a system that is amenable to experimental manipulation of sucrose content in the test genotype with inherently large variation for sucrose accumulation, as used in this study, will be more useful [14].

In line with the above contention, we have studied the transcriptomic changes associated with sucrose accumulation during natural ripening in three sugarcane genotypes with large difference in sucrose content. The sucrose accumulation in these genotypes were manipulated experimentally by externally applied ethylene to get a better resolution of the molecular changes associated with sucrose accumulation. As observed in commercial crop production there was considerable genotypic variation for ethylene-induced sucrose accumulation, with the greatest positive response occurring in LS genotype (Fig. 1). The exact reason for this variation is not clear but some molecular insights are emerging from the transcriptome analyses conducted in this study.

Analyses of transcriptomes from different genotypes and treatments have identified around 160,000 unigenes from a collection of > 3 billion transcripts generated in this study (Fig. 2; Additional file 11: Table S1). Out of these unigenes about 86,000 were annotated and grouped into three functional classes that are associated with general biological processes, cellular components and molecular functions. Within the biological process, unigenes associated with growth, metabolism, signaling, regulation, localization and tissue organization were enriched. This is not surprising considering the fact that the tissue studied was maturing stem segment which is a composite, functionally diverse organ with continued development of fibre and large vacuolar storage parenchyma. The tissue is also characterized by localised highly regulated transport, hydrolysis, synthesis and accumulation of sucrose in large amounts in parenchyma cells [6, 7, 12, 17, 31]. In the cellular component category, macromolecules, membranes, cell compartment and organelles were the dominant terms associated with the unigenes, while those classified in the molecular function grouping were mostly related to catalytic activity, transport, transcription factors, macromolecular processes and signal transduction. The dominance of these functional groups of unigenes in these broad categories again suggests the structural and functional specification occurring in the maturing sugarcane internodes distinguished by the progressively intensifying sucrose accumulation process. For instance, KEGG pathway analysis of a sub-set of unigenes relate them to carbohydrate metabolism, signal transduction and translation with each group containing over 1700 entries. These results strengthen the value of the dataset generated here for studying sucrose accumulation. It is important to note that in previous studies [13, 14, 41, 42], the vast majority of transcripts identified could not be assigned to functions associated with sucrose accumulation. In a more recent comparative analysis of gene expression between high- and low-sugar varieties, Thirugnanasambandam et al. (2017) [8] have identified 50–70 sugar/sucrose-related differentially expressed sugarcane transcripts but with large variation for expression occurring only for very few genes.

In this study, genotype, not the ethylene treatment, accounted for most of the differential gene expression observed. This gene expression pattern tallied well with the genotypic difference in sucrose accumulation. However, the underlying genetic elements contributing to the observed difference in gene expression remain unknown. For instance, a comparative study of differential expression of carbohydrate metabolism-related genes during stem development in a South African commercial sugarcane variety N19 (high-sugar) and the two ancestral species of commercial sugarcane, *Saccharum officinarum* and *S.robustum*, showed that transcript abundance varied most between commercial variety and their ancestors than between high-sugar (*S. officinarum*) and low-sugar (*S. robustum*) genotypes [14]. There is very little published information on comparative transcriptomics of sugarcane genotypes with large variation for sugar content. However, there is substantial variation for stem morphology and structure, especially for fibre content [43, 44], exists among sugarcane varieties, and this may, at least in part, explain the large genotypic variation for transcriptomes observed here. Further, as the main assimilated carbon of photosynthesis, sucrose plays a central role as energy molecule with different regulatory roles in a wide range of physiological processes [15, 45–48], influencing the activity of numerous growth and developmental gene networks, which are genotype-specific. This inherent genotype-dependent variation for developmental gene activity induced by sugars may also have contributed to the large genotypic variation for gene expression detected in this study.

The results presented here showed that sucrose and starch metabolism genes are expressed more abundantly in high-sugar genotypes than in the low-sugar clone. Interestingly, these genes were more responsive to ethylene treatment in low-sugar genotype (Additional file 10: Figure S10), suggesting the involvement of a complex higher order, hormone-regulated carbon source-sink interaction in ripener-induced sucrose accumulation. This is also giving more credentials to the proposal [14] that addressing sucrose accumulation in sugarcane from a source-sink regulation perspective may more likely to unravel the molecular regulatory mechanism(s) underpinning the phenomenon than by just comparing the changes in gene activity in high- and low-sugar clones in isolation. The enzymology of sucrose biosynthesis, and to some extent sucrose accumulation, is well established [2, 29–32, 38, 39, 49]. Sucrose, when reaches the stem, it is hydrolysed by sucrose synthase (SuSy) or one of the three invertases (soluble acid invertase, cell wall-bound acid invertase, neutral invertase) for driving various cell growth and developmental processes, and for storage. For sucrose accumulation, the hexoses produced from sucrose hydrolysis are used to resynthesize sucrose by sucrose phosphate synthase (SPS) and sucrose phosphatases (SPase). Sucrose transporters (SuTs), ATPase and a number of other enzymes are also involved in this process [2]. The GO annotation of DEGs showed high enrichment of genes associated with hormone signal transduction, macromolecular processes, transport, catalytic activity, transcription factors, phospohorylation, stress responses, starch and sugar metabolism, and plant-pathogen interaction. This GO annotation data broadly suggest the operation of a complex network of genes predominantly associated with cell wall synthesis, carbohydrate metabolism and biotic/abiotic stress responses, all possibly co-ordinately regulated by hormonal mechanism(s) that is yet to be described. To gain more insights into the regulation of sucrose accumulation, DEGs involved in carbohydrate metabolism were selected for pairwise comparison between high-and low-sugar clones with and without ethylene treatments. This analysis clearly shows a relatively high expression of several starch and sucrose metabolism pathway genes, and phloem sucrose loading and cell wall modifying genes in high-sugar genotype than in low-sugar cones. Though such clear distinction of these genes were not evident in some of the previous studies [13], this trend was reported recently by Thirugnansambandam et al. (2017) [8]. But what is more interesting is the finding that expression of both cytosolic acid invertase and cell wall invertase were upregulated in both high- and low-sugar genotypes but the expression of cell wall invertase genes was further boosted by ethylene treatment in low-sugar clone. Parallel to this, expression of ATPases were also induced by ethylene in low-sugar genotype. Acid-invertase activity is strongly co-related with growth and is reported to be regulated by an auxin-sugar control system with auxin stimulating and glucose suppressing the production of invertase [50]. Earlier reports showed a reduction in auxin content and accumulation of abscisic acid (ABA) in sugarcane during ripening [51] and tiller senescence [52]. In a recent study investigating ethylene-induced hormonal responses at the onset of sugarcane ripening, Cunha et al. (2017) [26] found no significant changes in total auxin content but an upregulation of auxin conjugation in ripening internodes of sugarcane in response to ethylene treatment and proposed that ethylene-mediated IAA inactivation inhibits growth and increases sucrose accumulation. They also reported a remarkable increase in ABA content and activity of neutral invertase, and a reduction in soluble acid invertase activity in maturing stems treated with ethylene compared to control. In our study, the expression of cell wall-bound invertases were increased following ethylene treatment in low-sugar genotype. Extracellular invertases are the key enzymes of apoplastic phloem unloading and they catalyse the hydrolysis of sucrose released into the apoplast. This mechanism accounts for long-distance sucrose transport, provides the substrate for growth, generates metabolic signals to control primary metabolism and defence responses, and supplies carbohydrates to sink tissues for storage [53]. ABA strongly induces extracellular invertases [53] and that is one of the mechanisms involved in stress-induced extracellular invertase production in plants. Further, ABA production during sugarcane ripening is genotype-dependent [51]. It is thus likely that the low-sugar genotype may be more responsive to ethylene-mediated ABA production than high-sugar clone, resulting in increased apoplastic invertase activity, increased sink strength and sugar accumulation [54] in response to ethylene.

Analysis of DEGs by MapMan classified them into several groups with the most enriched groups were associated with transcriptional regulation, ERFs, WRKYs, NAC domain transcription factors and hormone metabolism and signaling (Fig. 6a). Assessment of ERF expression indicated increased activity of ERFs in HS than in LS genotype in control, but the relative increase in ERF expression following ethylene treatment was remarkably higher in LS genotype making it more responsive to ethylene than HS clone. ERF family of genes play multiple roles in the regulation of plant metabolism, biotic and abiotic stress responses and growth and development [55]. ERF transcriptionally regulates accumulation of sucrose possibly through the increased production and sensitivity to ABA as observed in rice [56]. Increased ethylene activity is associated with lignin and fibre production, which is a critical component of assimilated carbon utilization, source-sink regulation, control of photosynthesis and ultimately sucrose accumulation. Ethylene-induced upregulation of NAC and WRKY transcription factors and ERFs suggest the interplay of ethylene and ABA in sucrose production and accumulation in sugarcane [57]. Clearly the enrichment of these genetic elements in DEGs indicated involvement of factors controlling biotic and abiotic stress responses in sucrose accumulation process [58]. However, a study on the role of drought-response genes in sucrose accumulation in sugarcane showed that while there were changes in stress-related genes in maturing stem tissue during sucrose accumulation, different sets of genes responded to water deficit and with increasing osmotic pressure occurring during sucrose storage. This example illustrates the complexity of molecular regulation of sucrose production and its accumulation in this sugar crop. From the results presented here it appears that decoding the ethylene- ABA- sugar signalling nexus may, at least in part, unravel the mechanism(s) underlying source-sink regulation and sucrose accumulation in sugarcane.

## Conclusion

**Fig. 7:**
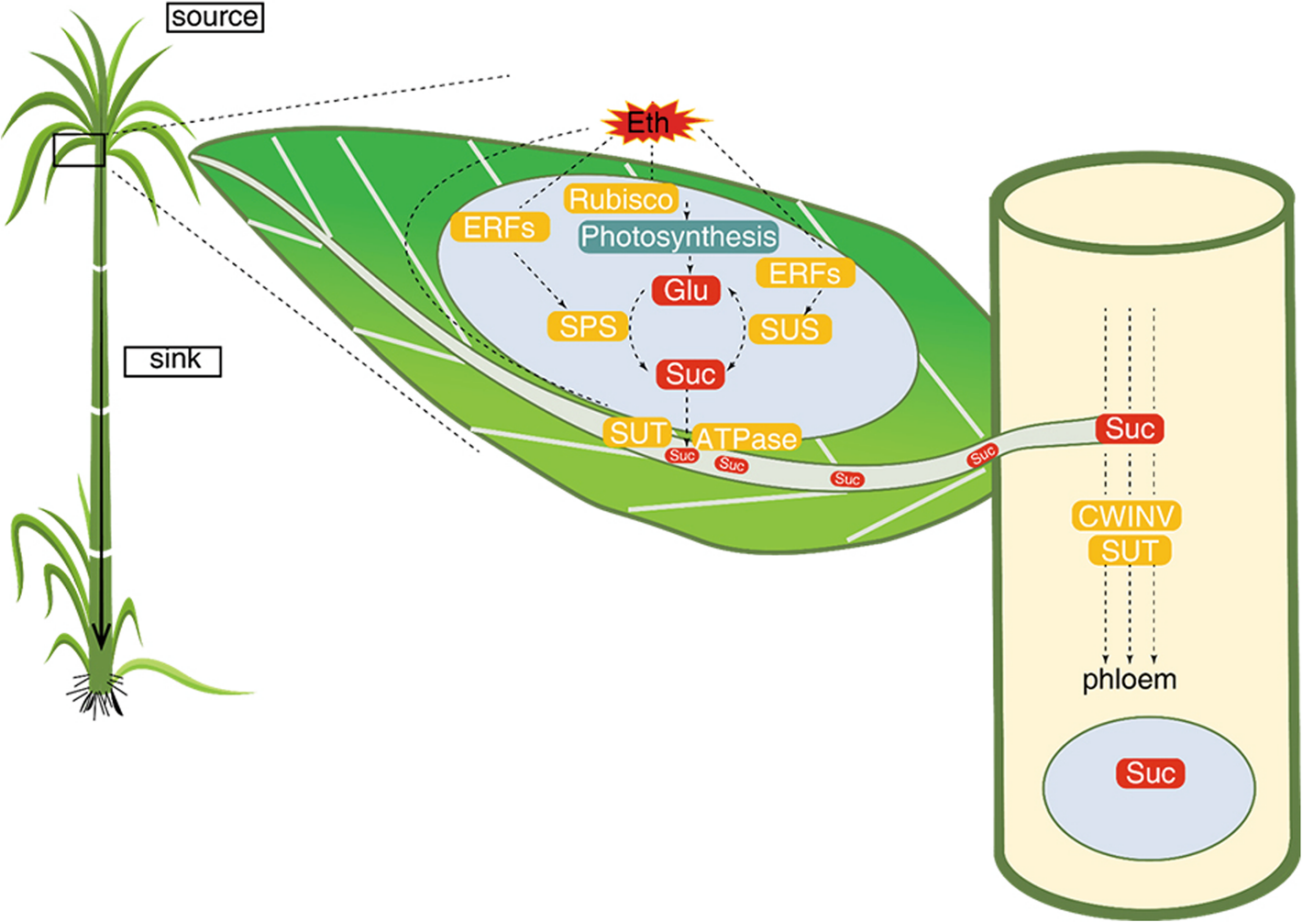
Schematic presentation of ethylene-regulated sugar production and translocation in sugarcane. The identified ethylene-regulated are integrated into a functional network of sugar production (source), sugar transportation and sugar aaccumulation (to sink). When leaves were treated with ethylene (Eth), Rubisco activity was enhanced; sucrose synthase (SUS), invertases (INV) and sucrose phosphate synthase (SPS) were up-regulated by translational regulation by ERFs, stimulating sucrose biosynthesis. At this time, sucrose and glucose could be acting as regulatory signals to control photosynthesis. After synthesis, sucrose will be transported from leaf to phloem through plasmodesmata (by sugar transporter, SUT - the symplastic route) and cell wall (by cell wall invertase- the apoplastic route). Then, sucrose was unloaded at the sink mainly through SUT. Sugar translocation from the leaves to phloem creates a sucrose gradient that facilitates the biochemical balance of sugar synthesis at the source

Molecular studies on sucrose accumulation based on cataloguing differentially expressed genes in high-and low-sugar genotypes generated a large volume of useful data but did not significantly advanced our mechanistic understanding of this phenomenon. Here we examined the transcriptomes of three different genotypes with large variation for sugar content and altered their sugar levels using a ripener, ethylene, to get a better resolution of the molecular changes associated with sucrose accumulation. In this study about 25,000 differentially expressed genes associated with ethylene response and sucrose accumulation were identified. It appears that genotype had a dominant effect on differential gene expression than ethylene treatment. This genotypic difference is possibly arising from the significant inherent developmental and structural variation, and the variation for sugar signaling gene networks occurring in different genetic backgrounds. Not surprisingly sucrose and starch metabolism genes were more abundantly expressed in high-sugar genotypes compared to low-sugar clones. However, low-sugar genotype was more responsive to ethylene-induced gene expression and sucrose accumulation. An interplay of ethylene, ABA and sugars may be modulating sucrose production and accumulation in sugarcane. Ethylene, through its preferential activation of genes associated with carbohydrate metabolism, such as apoplastic invertases, SuSy, etc., probably increased the sink strength allowing more phloem unloading and accumulation of sucrose in storage parenchyma in ethylene-treated low-sugar genotype. Though this framework is presented as a simple model for testing, regulation of source and sink activities to sustain growth and development, and then storage of reserve food, will undoubtedly involve a multitude of mechanisms operating at different levels of organization, spanning from membrane transport to crop-level photosynthesis. We speculate that a nexus of a few master regulators, possibly under hormonal control, operating at a higher order may be controlling this developmental process (Fig. 7).

## Methods

### Plant materials, ethylene treatment and tissue sampling for transcriptomics and sugar analysis

Three sugarcane genotypes with different sugar content were used for this study. They are ROC22, a high-sugar commercial variety (HS; sucrose content 15.0%), YT71–210, a moderate-sugar non-commercial genotype ((MS; sucrose content 12.6%) and GT86–887, a low-sugar non-commercial genotype (LS; sucrose content 6.0%). They were selected because of their similar growth habit and biomass production but with large differences in sugar content. Setts (stem cuttings with 2–3 buds) of these clones were obtained from disease-free plants from propagation plots and planted in the experimental farm of Sugarcane Research Institute, Nanning, China (SRI) in February 2016. They were grown till October 2016 following the best crop management practices recommended by SRI. The crops received nitrogen (360 kg/ha) fertiliser and was rain-fed with supplementary irrigation when required. For all treatments the experimental unit for each clone was a 5 × 7 m rows plot with 1.2 m inter-row spacing, and they were replicated thrice. Plants were treated with 400 mg/L ethylene solution (prepared from Ethephon, a commercial preparation containing 40% 2-chloroethyl phosphonic acid in aqueous solution) or water as foliar spray till run-off from the lamina in mid-October 2016. Plants were 8-month-old and in the early stage of sucrose accumulation when treated with ethephon. For sucrose analyses, whole stems were sampled just before the treatment and on day 3, 5 and 7 following ethylene treatment. And sucrose content (three biological replicates each with three technical replicates for each treatment for all four time points) was determined following the protocols described previously [59]. For transcriptome analyses, developing stalk tissues, 20 cm above the node attached to the 2nd youngest fully expanded leaf, which is highly photosynthetically active, were sampled on day 7 following ethylene treatment. All samples were flash frozen in liquid nitrogen and processed for transcriptome analyses (two biological replicates each with three technical replicates for each treatment for day 7 following ethylene treatment) In October 2016 the maximum-minimum temperature in the Experimental Farm were 36 and 16 °C with 63–90%, relative humidity.

### RNA isolation and sequencing

Total RNA was extracted from frozen samples using the Qiagen RNA plant mini-kit (Qiagen, Hilden, Germany) following the manufacturer’s instructions. The quality and quantity of RNA were determined by Nanodrop and Qubit, respectively, and Agilent 2100 was used to evaluate the RNA Integrity, High quality RNA samples with Integrity Number > 8 were used for constructing cDNA libraries and sequencing using Illumina HiSeq 2000 platform (Beijing Novogene Bioinformatics Technology Co. Ltd., Beijing, China).

### Data processing, assembly and annotation

Clean sequence reads were obtained by removing all adaptor sequences, empty reads and low quality reads (Q < 30 and length < 50 bp) from raw reads. Clean reads were assembled by Trinity software package [27]. The longest transcript of each sub-component was defined as a unigene. All the assembled unigenes were searched against Nr (Non-redundant protein database), Nt (Non-redundant nucleotide sequences), and SwissProt, using the NCBI blast 2.2.28 with a E-value cut-off of 10^− 5^ and KOG/COG (Eukaryotic Orthologous Group/ Clusters of Orthologous Groups of proteins) database with an E-value cut-off of 10^− 3^. PFAM protein family alignments were performed using the HMMER 3.0 package with an E-value cut-off of 10^− 2^. Gene ontology (GO) classification of each gene model was carried out by Blast2GO v2.5 with an E-value of 10^− 6^ [60] and KEGG classification was performed using KASS and the KEGG (Kyoto Encyclopedia of Genes and Genomes) Automatic Annotation Server with a E-value of 10^− 10^ [61].

### Analysis of gene expression patterns

The unigenes obtained were used to assemble into Ref and the clean reads were aligned to the Ref by RSEM [62]. The abundance of all genes was normalized and calculated using uniquely mapped reads by the FRKM method. The differential expression analysis was performed using DEGseq method with the threshold q-value of < 0.005 and Fold Change of > 2 demarcating significantly different expression levels [63]. GO and KO enrichment analyses were performed based on the identified DEGs. GO-enrichment analyses were carried out using the GPseq method based on the Wallenius non-central hypergeometric distribution with *P*-value of < 0.05 [64]. Enriched GO terms in DEGs were checked and compared to the genome background. KEGG pathway enrichment analysis of DEGs was carried out using KOBAS, and the enriched pathways were also compared with the genome background based on the hypergeometric distribution [65].

### Expression analysis of genes involved in sugar metabolism

The expression of sugar biosynthesis and metabolism genes in response to ethylene treatment in tissues of all three test genotypes were studied using quantitative real-time PCR (qRT-PCR). The qRT-PCR primers were designed with the Primer 3.0 software (http://biotools.umassmed.edu/bioapps/primer3_www.cgi), and the sequences used for primer design are listed in Additional file 15: Table S5. qRT-PCR was performed in the ABI StepOne™ Plus Real-Time PCR System with the SYBR Green PCR Master Mix (Takara), with three biological replicates for each gene and three technical repeats per experiment. Relative gene expression was normalized by comparison with the expression of sugarcane GAPDH (EF189713) [66], and analyzed using the 2-^ΔΔCT^ method [67].

## Additional files

Additional file 1: **Figure S1.** FPKM density distribution (A) and FPKM distribution box plot (B). CK- check (water control), T- ethephon treatment; HS, MS and LS are high-sugar, medium-sugar and low-sugar sugarcane genotypes, respectively. (JPG 1608 kb)

Additional file 2: **Figure S2.** The distribution of transcripts and unigenes assembled from the RNA-seq data. (JPG 3542 kb)

Additional file 3: **Figure S3.** Gene annotation of unigenes obtained from RNA-seq data. (JPG 1241 kb)

Additional file 4: **Figure S4**. Gene expression pattern analysis in different tissue samples. CK- check (water control), T- ethephon treatment; HS, MS and LS are high-sugar, medium-sugar and low-sugar sugarcane genotypes, respectively. (JPG 2560 kb)

Additional file 5: **Figure S5.** DEG enrichment analysis by pairwise comparisons. The three GO categories are biological processes (A), molecular function (B), and cellular components (C). The significance of the most represented GO Slims in each comparison pair is indicated using log-transformed *P*-value (red); the dark gray areas represent missing values. CK- check (water control), T- ethephon treatment; HS, MS and LS are high-sugar, medium-sugar and low-sugar sugarcane genotypes, respectively. (JPG 4487 kb)

Additional file 6 **Figure S6.** KEGG pathways that were significantly enriched in pairwise comparisons. The significance of the most strongly represented pathway in each comparison pair is indicated using log-transformed *P*-value (red); the dark gray areas represent missing values. CK- check (water control), T- ethephon treatment; HS, MS and LS are high-sugar, medium-sugar and low-sugar sugarcane genotypes, respectively. (JPG 2006 kb)

Additional file 7: **Figure S7.** Differentially expressed genes involved in starch and sucrose metabolism HS_CK and LS_CK comparison. HS and LS are high-and low-sugar sugarcane genotypes, respectively. CK- check (control). (JPG 2764 kb)

Additional file 8: **Figure S8.** Comparison of differentially expressed photosynthetic genes in HS_CK and LS_CK. HS and LS are high- sugar and low-sugar sugarcane genotypes, respectively. CK- check (control). (JPG 3132 kb)

Additional file 9: **Figure S9.** Transcription pattern of candidate genes analyzed by quantitative real-time PCR. ROC22, YT71–210 and GT86–887 are high-sugar, medium-sugar and low-sugar sugarcane genotypes, respectively, used in the study. (JPG 1925 kb)

Additional file 10: **Figure S10.** Differentially expressed genes involved in starch and sucrose metabolism between LS_T and LS_CK**.** LS: low-sugar sugarcane genotype, T: ethephon treatment, CK: control (water). (JPG 151 kb)

Additional file 11: **Table S1.** Key statics of sugarcane developing stem tissue-derived RNA-seq data used in the study. (PDF 59 kb)

Additional file 12: **Table S2.** Frequency of transcripts and unigenes of different length identified from RNA-seq data. (PDF 45 kb)

Additional file 13: **Table S3.** Statistics of transcripts and unigenes assembled from RNA-seq data. (PDF 44 kb)

Additional file 14: **Table S4.** Annotation statistics of the unigenes assembled from RNA-seq data. (PDF 48 kb)

Additional file 15: **Table S5.** Primers used for Real time PCR. (PDF 53 kb)

## Data Availability

The data discussed in this publication have been deposited in NCBI’s Gene Expression Omnibus [68] and are accessible through GEO Series accession number GSE130757 (https://www.ncbi.nlm.nih.gov/geo/query/acc.cgi?acc=GSE130757).

## Abbreviations

ABA: Abscisic acid
COG: Clusters of Orthologous Groups
DEGs: Differentially expressed genes
ERFs: Ethylene responsive transcription factors
GAPDH: Glyceraldehyde phosphate dehydrogenase
GO: Gene Ontology
HSF: Heat shock factor
KAAS: KEGG Automatic Annotation Server
KEGG: Kyoto Encyclopedia of Genes and Genomes
KOG: EuKaryotic Orthologous Group
NR: NCBI non-redundant database
NT: Non-redundant nucleotide sequences
SPase: Sucrose phosphatases
SPS: Sucrose phosphate synthase
SuSy: Sucrose synthase
SuTs: Sucrose transporters
TFs: Transcription factors
