# Supplementary figures and images for: Ethylene-mediated improvement in sucrose accumulation in ripening sugarcane involves increased sink strength
Source: BMC Plant Biol. 2019 Jun 28;19:285. doi: 10.1186/s12870-019-1882-z (PMC6599285; doi:10.1186/s12870-019-1882-z)

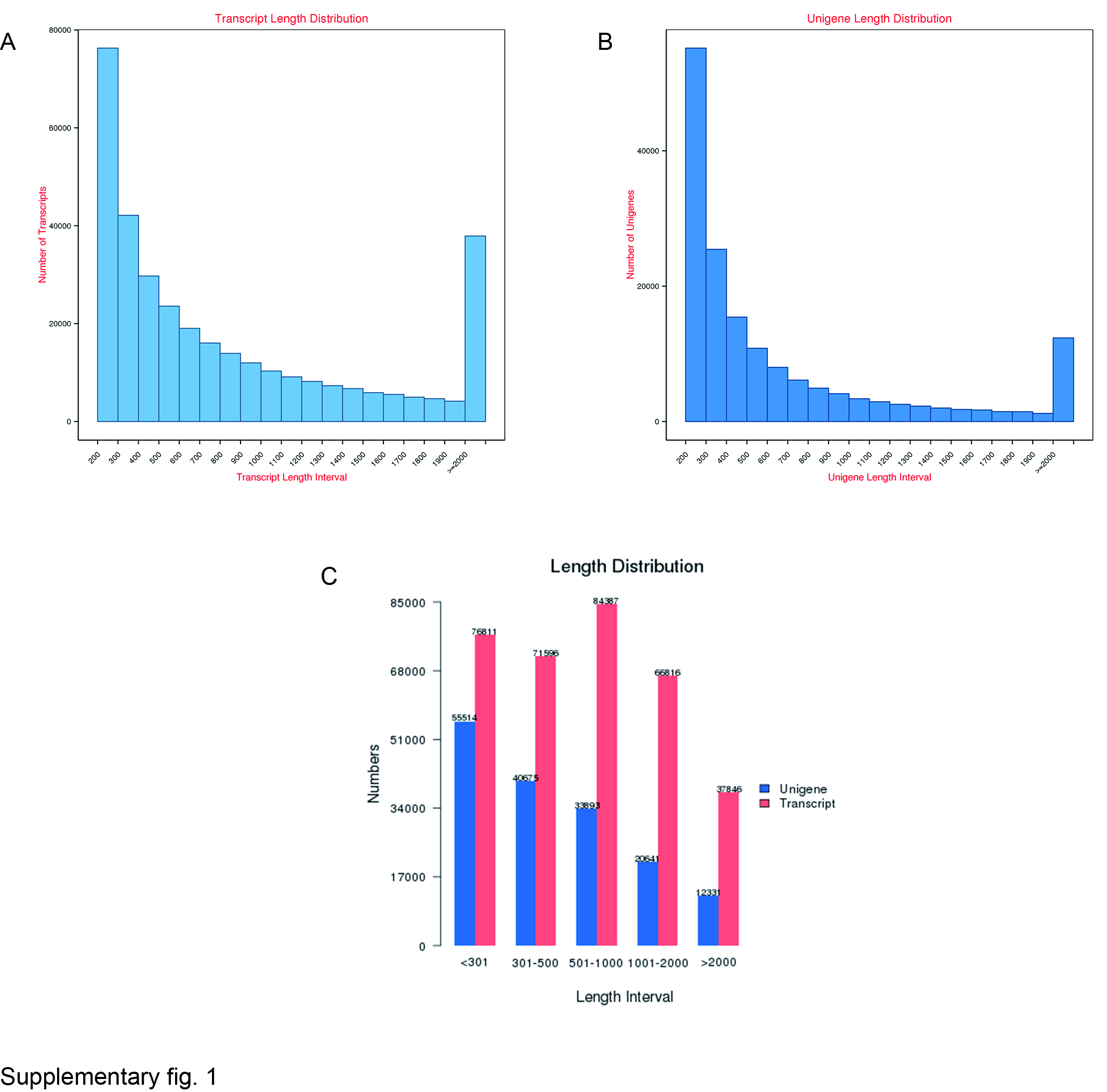

Supplement: Supplementary file 1 — Figure S1. FPKM density distribution (A) and FPKM distribution box plot (B). CK- check (water control), T- ethephon treatment; HS, MS and LS are high-sugar, medium-sugar and low-sugar sugarcane genotypes, respectively. (JPG 1608 kb) [file 12870_2019_1882_MOESM1_ESM.jpg]

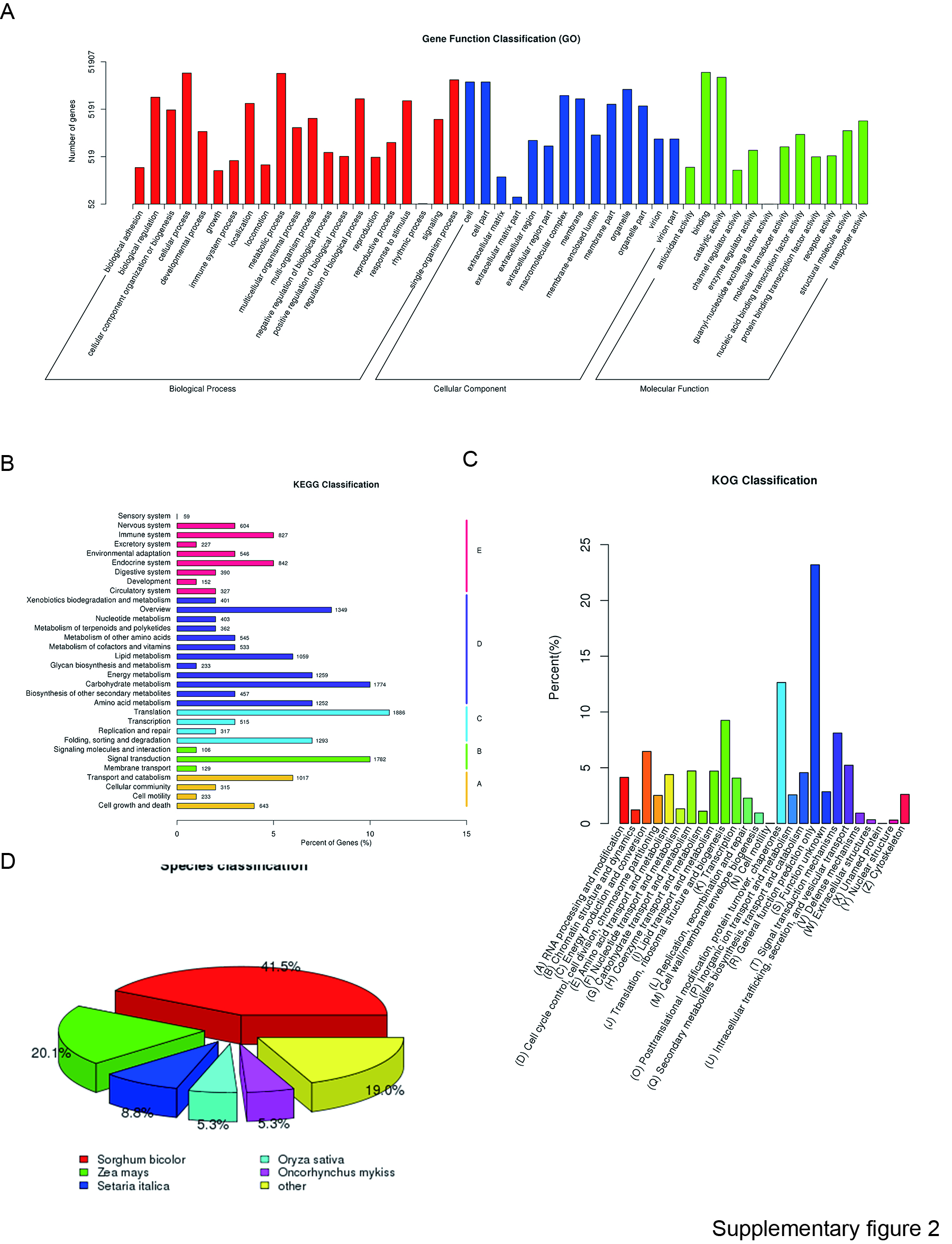

Supplement: Supplementary file 2 — Figure S2. The distribution of transcripts and unigenes assembled from the RNA-seq data. (JPG 3542 kb) [file 12870_2019_1882_MOESM2_ESM.jpg]

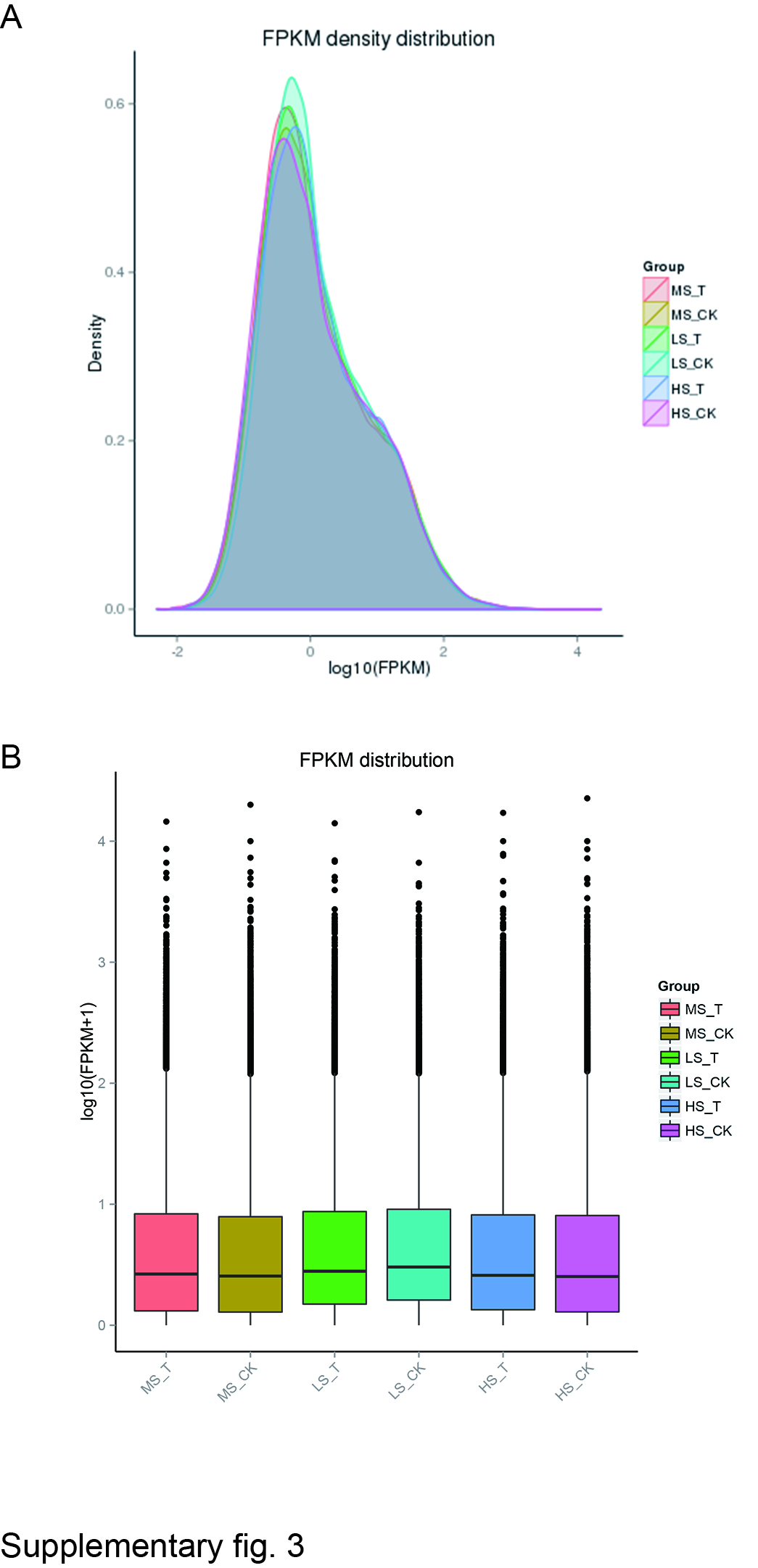

Supplement: Supplementary file 3 — Figure S3. Gene annotation of unigenes obtained from RNA-seq data. (JPG 1241 kb) [file 12870_2019_1882_MOESM3_ESM.jpg]

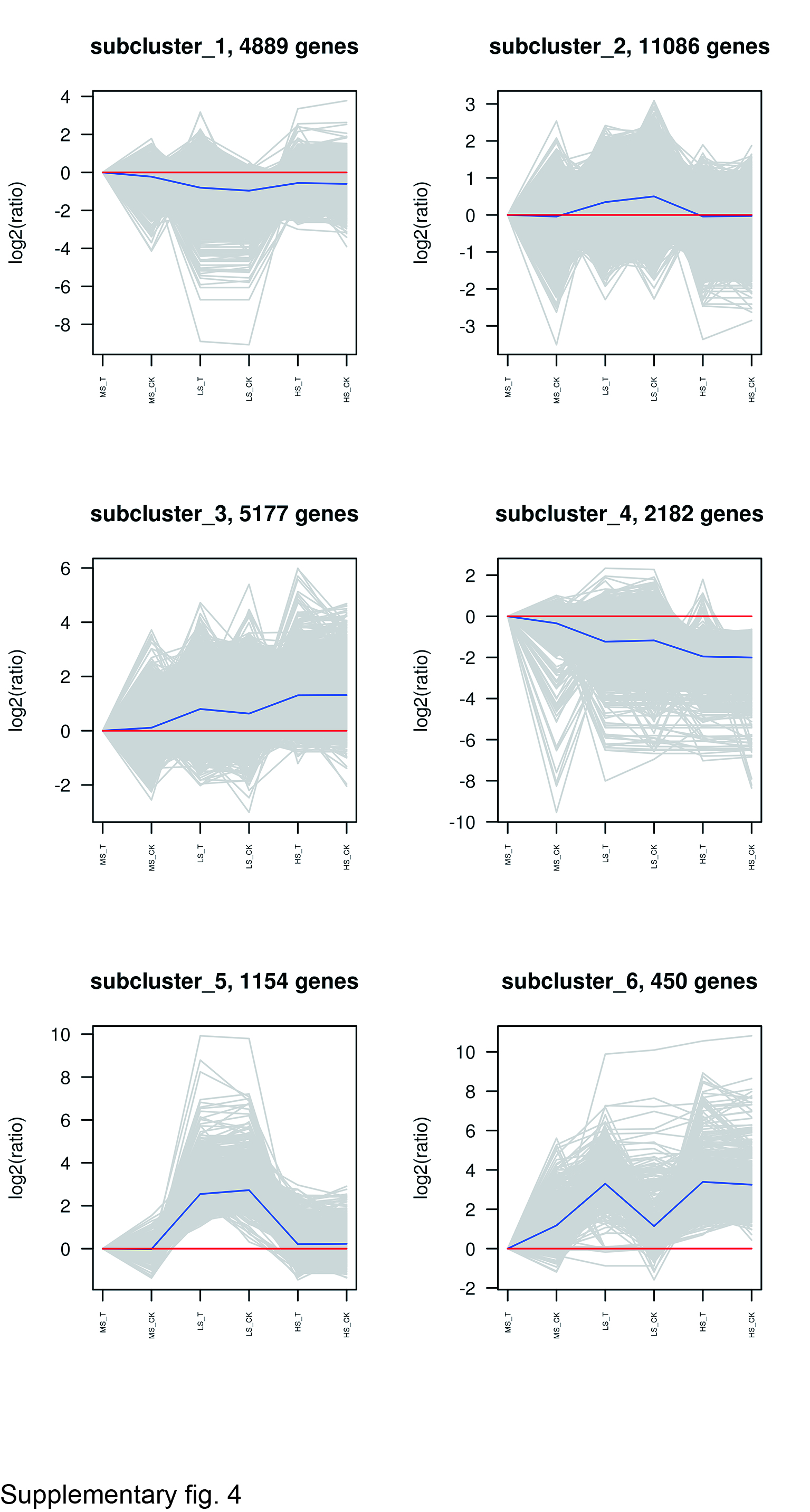

Supplement: Supplementary file 4 — Figure S4. Gene expression pattern analysis in different tissue samples. CK- check (water control), T- ethephon treatment; HS, MS and LS are high-sugar, medium-sugar and low-sugar sugarcane genotypes, respectively. (JPG 2560 kb) [file 12870_2019_1882_MOESM4_ESM.jpg]

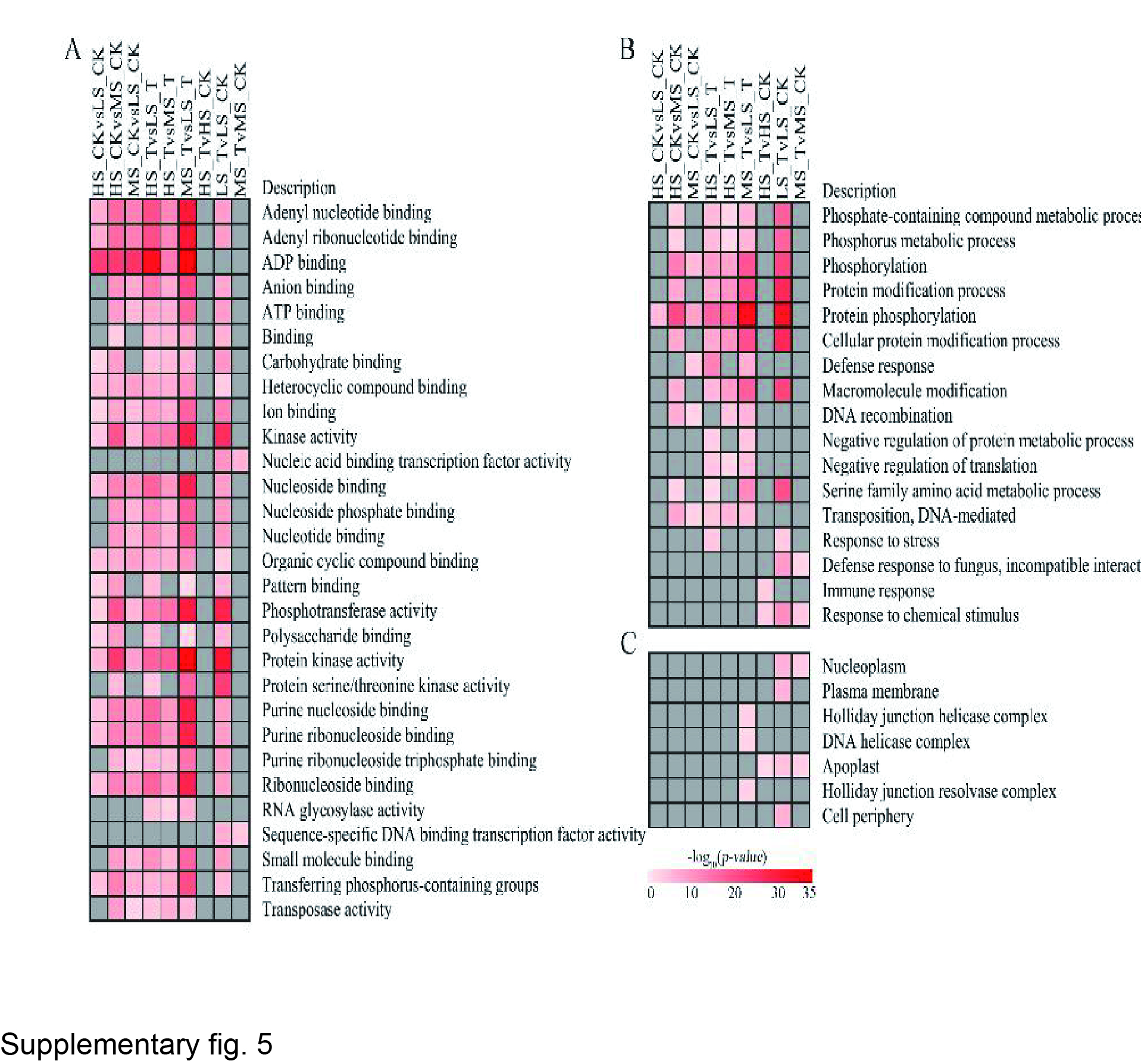

Supplement: Supplementary file 5 — Figure S5. DEG enrichment analysis by pairwise comparisons. The three GO categories are biological processes (A), molecular function (B), and cellular components (C). The significance of the most represented GO Slims in each comparison pair is indicated using log-transformed P-value (red); the dark gray areas represent missing values. CK- check (water control), T- ethephon treatment; HS, MS and LS are high-sugar, medium-sugar and low-sugar sugarcane genotypes, respectively. (JPG 4487 kb) [file 12870_2019_1882_MOESM5_ESM.jpg]

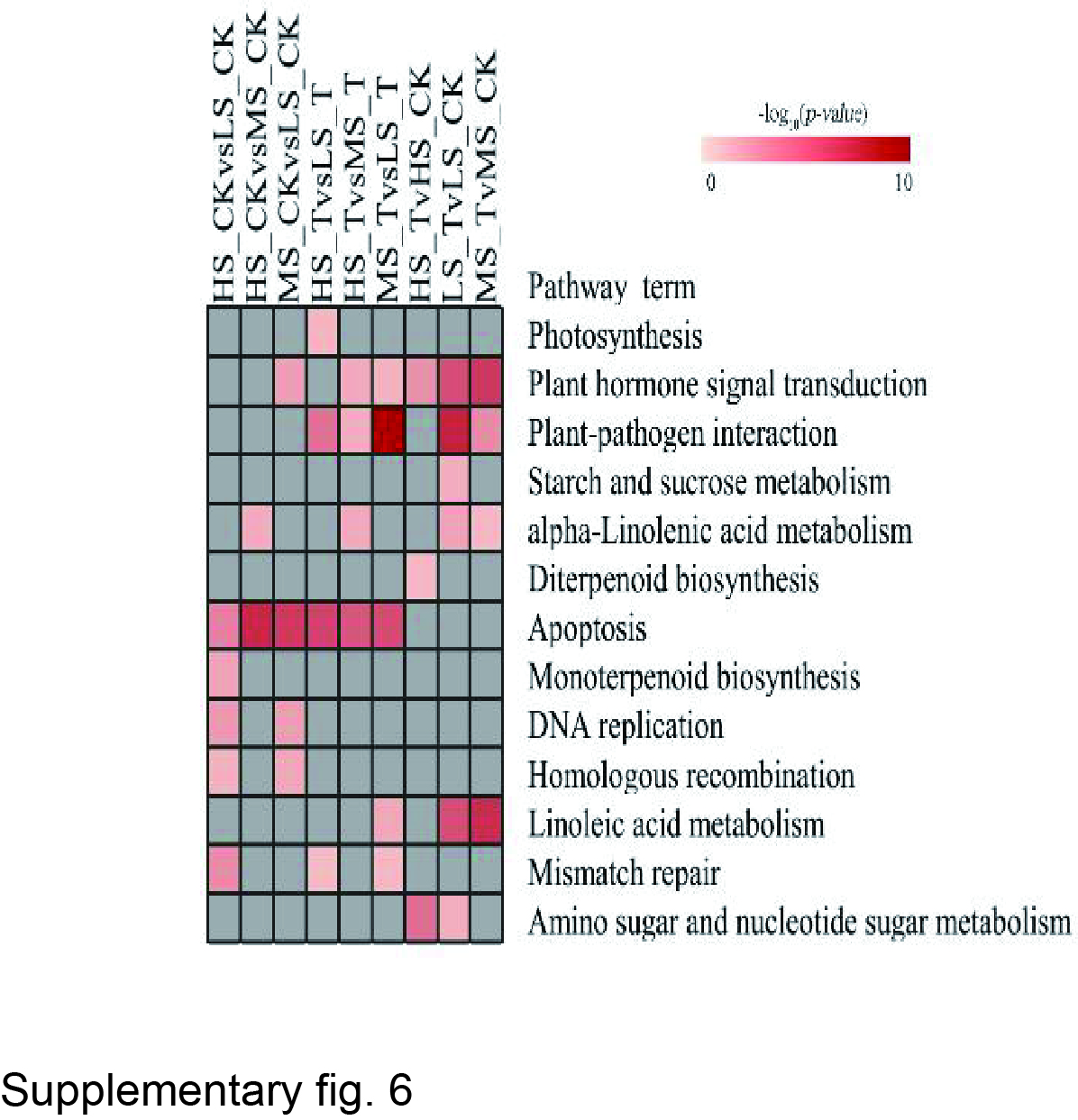

Supplement: Supplementary file 6 — Figure S6. KEGG pathways that were significantly enriched in pairwise comparisons. The significance of the most strongly represented pathway in each comparison pair is indicated using log-transformed P-value (red); the dark gray areas represent missing values. CK- check (water control), T- ethephon treatment; HS, MS and LS are high-sugar, medium-sugar and low-sugar sugarcane genotypes, respectively. (JPG 2006 kb) [file 12870_2019_1882_MOESM6_ESM.jpg]

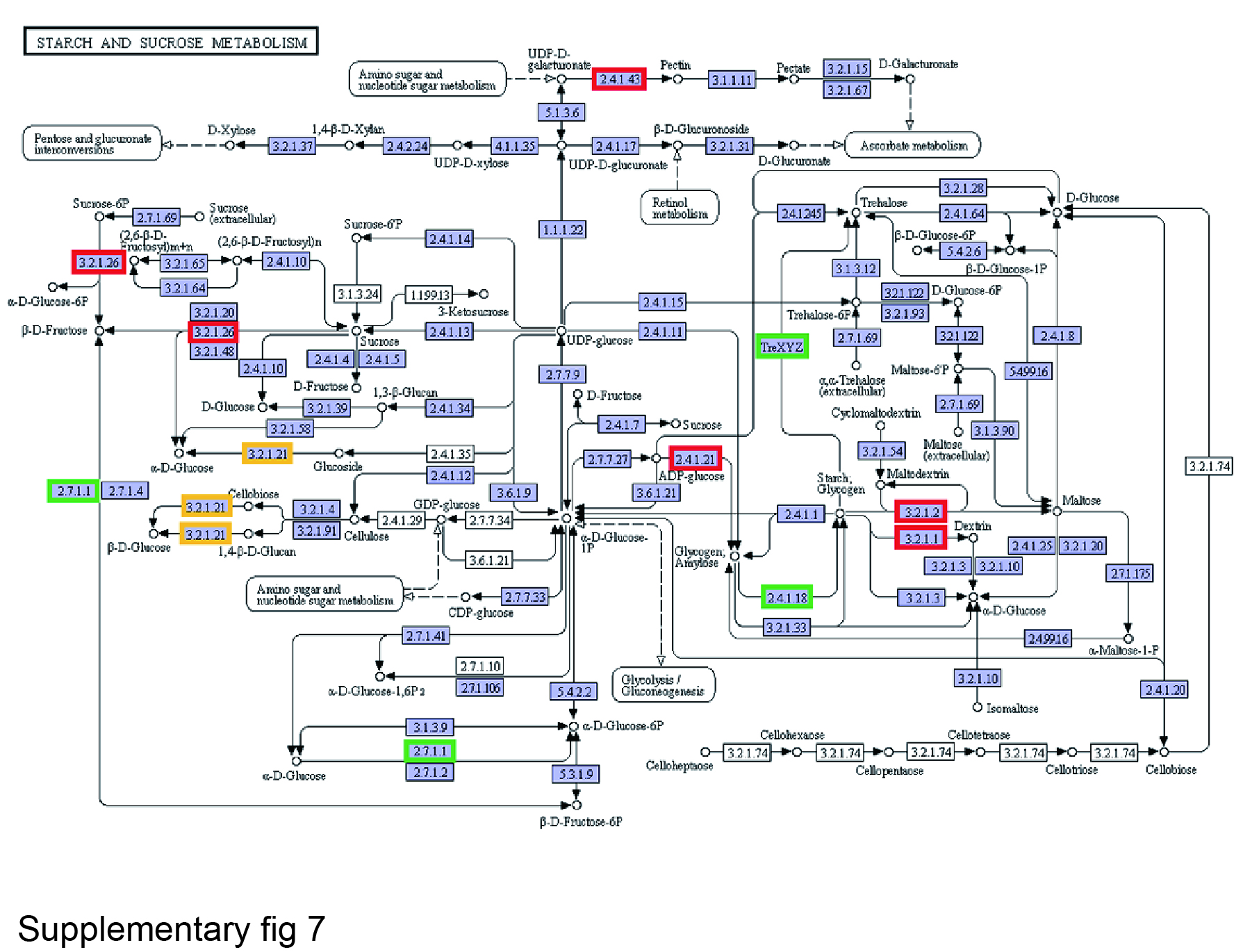

Supplement: Supplementary file 7 — Figure S7. Differentially expressed genes involved in starch and sucrose metabolism HS_CK and LS_CK comparison. HS and LS are high-and low-sugar sugarcane genotypes, respectively. CK- check (control). (JPG 2764 kb) [file 12870_2019_1882_MOESM7_ESM.jpg]

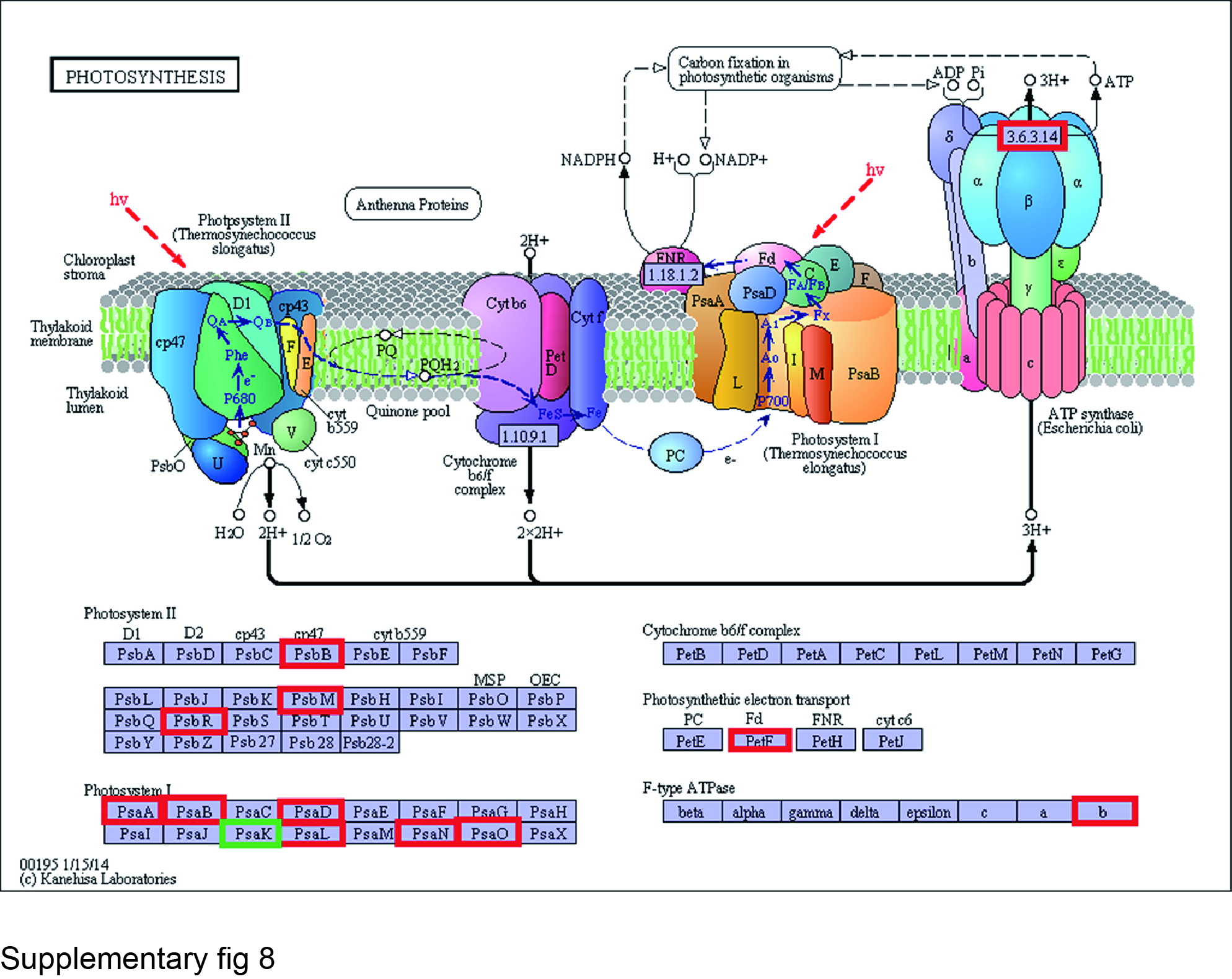

Supplement: Supplementary file 8 — Figure S8. Comparison of differentially expressed photosynthetic genes in HS_CK and LS_CK. HS and LS are high- sugar and low-sugar sugarcane genotypes, respectively. CK- check (control). (JPG 3132 kb) [file 12870_2019_1882_MOESM8_ESM.jpg]

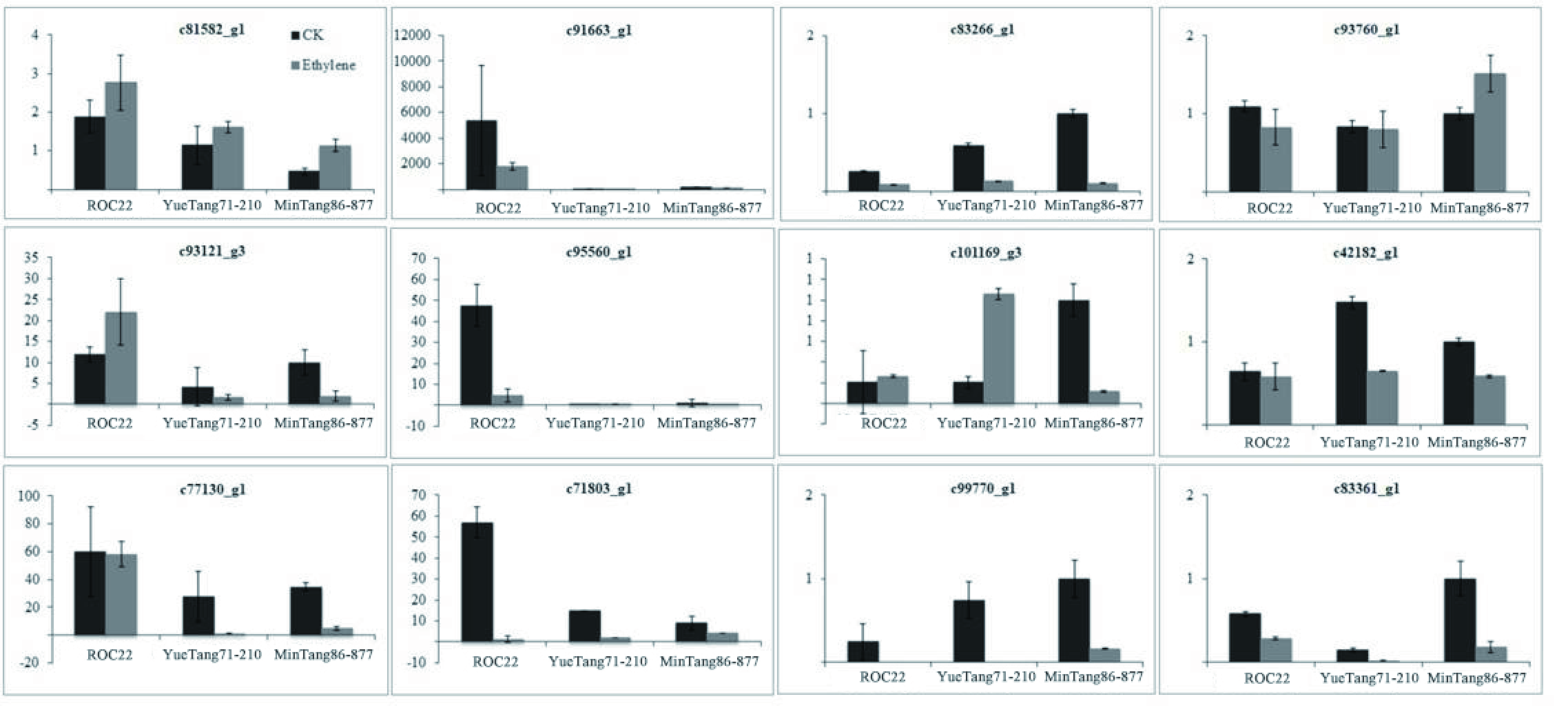

Supplement: Supplementary file 9 — Figure S9. Transcription pattern of candidate genes analyzed by quantitative real-time PCR. ROC22, YT71–210 and GT86–887 are high-sugar, medium-sugar and low-sugar sugarcane genotypes, respectively, used in the study. (JPG 1925 kb) [file 12870_2019_1882_MOESM9_ESM.jpg]

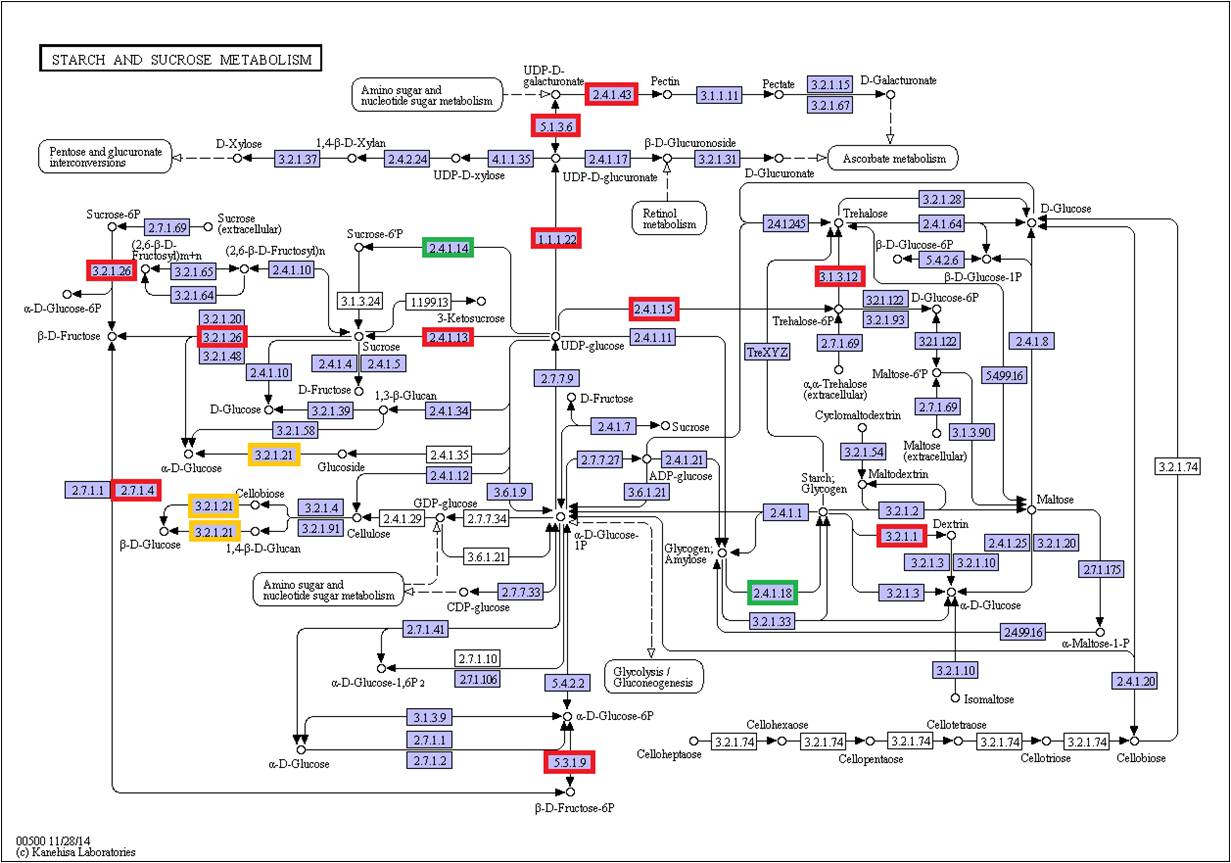

Supplement: Supplementary file 10 — Figure S10. Differentially expressed genes involved in starch and sucrose metabolism between LS_T and LS_CK. LS: low-sugar sugarcane genotype, T: ethephon treatment, CK: control (water). (JPG 151 kb) [file 12870_2019_1882_MOESM10_ESM.jpg]
