# Supplementary material for: Ethylene-mediated improvement in sucrose accumulation in ripening sugarcane involves increased sink strength
Source: BMC Plant Biol. 2019 Jun 28;19:285. doi: 10.1186/s12870-019-1882-z (PMC6599285; doi:10.1186/s12870-019-1882-z)
Supplement: Supplementary file 11 — Table S1. Key statics of sugarcane developing stem tissue-derived RNA-seq data used in the study. (PDF 59 kb) [file 12870_2019_1882_MOESM11_ESM.pdf]

Supplementary Table 1. Key statics of sugarcane developing stem tissue-derived RNA-seq data used in the study

Sample	Raw Reads	Clean reads	Clean bases	Error (%)	Q20 (%)	Q30 (%)	GC (%)
MS_T1_1	31163840	30100751	3.76G	0.03	96.3	92.31	53.55
MS_T1_2	31163840	30100751	3.76G	0.04	93.52	87.96	53.52
MS_T2_1	42547794	40832307	5.1G	0.03	96.45	92.61	52.08
MS_T2_2	42547794	40832307	5.1G	0.04	93.55	87.98	52.03
MS_CK1_1	30125876	28789148	3.6G	0.03	96.53	92.76	54.04
MS_CK1_2	30125876	28789148	3.6G	0.04	94.16	89.06	54.03
MS_CK2_1	31541733	30095376	3.76G	0.03	96.6	92.9	54.12
MS_CK2_2	31541733	30095376	3.76G	0.04	93.65	88.23	54.11
LS_T1_1	35105070	33899692	4.24G	0.03	96.16	92.05	52.93
LS_T1_2	35105070	33899692	4.24G	0.04	93.36	87.64	52.91
LS_T2_1	29665898	28356704	3.54G	0.03	96.48	92.65	53.54
LS_T2_2	29665898	28356704	3.54G	0.04	93.64	88.03	53.47
LS_CK1_1	32532416	31345758	3.92G	0.03	96.24	92.21	53.08
LS_CK1_2	32532416	31345758	3.92G	0.04	93.99	88.72	53.05
LS_CK2_1	33588701	32446507	4.06G	0.03	96.14	92.01	53.67
LS_CK2_2	33588701	32446507	4.06G	0.04	93.62	88.08	53.64
HS_T1_1	33248563	31868529	3.98G	0.03	96.57	92.83	54.38
HS_T1_2	33248563	31868529	3.98G	0.04	94.21	89.12	54.37
HS_T2_1	30668558	29385779	3.67G	0.03	96.56	92.82	54.22
HS_T2_2	30668558	29385779	3.67G	0.04	94.26	89.22	54.21
HS_CK1_1	34669905	33328158	4.17G	0.03	96.6	92.91	53.74
HS_CK1_2	34669905	33328158	4.17G	0.04	94.31	89.3	53.73
HS_CK2_1	31892553	30695645	3.84G	0.03	96.6	92.85	55.12
HS_CK2_2	31892553	30695645	3.84G	0.04	94.18	89.06	55.11

Sample: Sample name: 1 is for the left reads and2 for the right reads. Clean reads stand for the left and right. T: ethehone treatment, CK: check (water); HS, MS and LS are high-sugar, medium-sugar and low-sugar sugarcane genotypes, respectively.
