# Supplementary material for: Ethylene-mediated improvement in sucrose accumulation in ripening sugarcane involves increased sink strength
Source: BMC Plant Biol. 2019 Jun 28;19:285. doi: 10.1186/s12870-019-1882-z (PMC6599285; doi:10.1186/s12870-019-1882-z)
Supplement: Supplementary file 12 — Table S2. Frequency of transcripts and unigenes of different length identified from RNA-seq data. (PDF 45 kb) [file 12870_2019_1882_MOESM12_ESM.pdf]

Supplementary Table 2. Frequency of transcripts and unigenes of different length identified from RNA-seq data

Transcript length interval	200-500 bp	500-1k bp	1-2k bp	>2k bp	Total
Number of transcripts	148407	84387	66816	37846	337456
Number of UniGenes	96189	33893	20641	12331	163054
