# Supplementary material for: Ethylene-mediated improvement in sucrose accumulation in ripening sugarcane involves increased sink strength
Source: BMC Plant Biol. 2019 Jun 28;19:285. doi: 10.1186/s12870-019-1882-z (PMC6599285; doi:10.1186/s12870-019-1882-z)
Supplement: Supplementary file 13 — Table S3. Statistics of transcripts and unigenes assembled from RNA-seq data. (PDF 44 kb) [file 12870_2019_1882_MOESM13_ESM.pdf]

Supplementary Table 3. Statistics of transcripts and unigenes assembled from RNA-seq data

	Minimum Length	Mean Length	Median Length	Maximum Length	N50	N90	Total Nucleotides
Transcripts	201	930	585	15657	1488	387	313757146
Unigenes	201	731	404	15657	1198	286	119150331
