# Supplementary material for: Ethylene-mediated improvement in sucrose accumulation in ripening sugarcane involves increased sink strength
Source: BMC Plant Biol. 2019 Jun 28;19:285. doi: 10.1186/s12870-019-1882-z (PMC6599285; doi:10.1186/s12870-019-1882-z)
Supplement: Supplementary file 14 — Table S4. Annotation statistics of the unigenes assembled from RNA-seq data. (PDF 48 kb) [file 12870_2019_1882_MOESM14_ESM.pdf]

Supplementary Table 4. Annotation statistics of the unigenes assembled from RNA-seq data

	Number of nigenes	Percentage (%)
Annotated in NR	67147	41.18
Annotated in NT	59657	36.58
Annotated in KO	17330	10.62
Annotated in SwissProt	37873	23.22
Annotated in PFAM	44386	27.22
Annotated in GO	51907	31.83
Annotated in KOG	19194	11.77
Annotated in all Databases	8385	5.14
Annotated in at least one Database	86944	53.32
Total Unigenes	163054	100
