# Supplementary material for: Ethylene-mediated improvement in sucrose accumulation in ripening sugarcane involves increased sink strength
Source: BMC Plant Biol. 2019 Jun 28;19:285. doi: 10.1186/s12870-019-1882-z (PMC6599285; doi:10.1186/s12870-019-1882-z)
Supplement: Supplementary file 15 — Table S5. Primers used for Real time PCR. (PDF 53 kb) [file 12870_2019_1882_MOESM15_ESM.pdf]

Supplementary Table5. Primers used for Real time PCR

Genes	Primers	Sequences
c81582_g1	Forward	CATGGGTGGCAGAGAAGC
	Reverse	CAAGGTGAACTTCCCCGAC
c91663_g1	Forward	GTTTTAGCTTAACCCTGCTGTC
	Reverse	GATTGGAGAGGGAAGAGAAGC
c93409_g3	Forward	GTCTGAAATTGCTCACGGTG
	Reverse	GAATTACACCTGGTCTGGTCAG
c93760_g1	Forward	GAAATGGTCGTGGTCTAGGG
	Reverse	GCTTCCTCGCCTACCACC
c77130_g1	Forward	CTTCCAAACGACTCAAGTCCCT
	Reverse	CACGACTCCTCTGCCCCTC
c84505_g3	Forward	GAGATCCTTGCCAAACTAACTG
	Reverse	GCAAAACCACTTGTTGTATTGAC
c78352_g3	Forward	CATTCCCGTCCATGAATCC
	Reverse	CGTATAATGCCACATCTGTGC
c87273_g1	Forward	GGGATCACCACGTAGTTCACC
	Reverse	CCTCAAGGTGCAGAACAGCC
c93121_g3	Forward	CCTTTCCTTGGTGAAGTATGGC
	Reverse	CAAAAGTGGACTGACCAAAGACC
c95560_g1	Forward	GAGCAGCATCAGTGTTGGAAG
	Reverse	GATATGACATGCTTGGTCGGAC
c101031_g1	Forward	AAGAAGAAGAAAGGAAGATGGTTG
	Reverse	AGGTAGAGTGGAAGGTATTGTTGG
c71803_g1	Forward	GCTTCCTTGCCTTGTGAATG
	Reverse	TTTAATTTGGTGCATCTGTATCG
c101169_g3	Forward	CATCCTCGCCACGGACTAC
	Reverse	GGGAATGACGGTGAACAGC
c83266_g1	Forward	ATCTTTGTTGGCCTTCTTCC
	Reverse	CTTGGCTGGCACTGTAATGTTC
c83361_g1	Forward	AAACATTGAAATACGCTACGGG
	Reverse	GCTGTGTGAACTCTGGTCTGAAC
c95985_g1	Forward	TTCTGGCGAGTGATAATGACC
	Reverse	TTTAACCCGCTGCCGATTC
c97708_g1	Forward	TTCATATTAAATAGGCACCAAAACG
	Reverse	CCATCTCATGTGACCTGTAGGC
c42182_g1	Forward	TTGGTGAAGATGTTAGTGGAATG
	Reverse	GAGAAGTTCAATCCATTTGTCAG
